# CA125 and age-based models for ovarian cancer detection in primary care: a population-based external validation study

**DOI:** 10.1038/s41416-025-03165-4

**Published:** 2025-09-17

**Authors:** Kirsten D. Arendse, Fiona M. Walter, Gary Abel, Brian Rous, Willie Hamilton, Emma J. Crosbie, Garth Funston

**Affiliations:** 1 Wolfson Institute of Population Health, Queen Mary University of London Barts and The London School of Medicine and Dentistry, London, United Kingdom; 2 Primary Care Unit, Department of Public Health and Primary Care, University of Cambridge, Cambridge, United Kingdom; 3 University of Exeter Medical School, University of Exeter, Exeter, United Kingdom; 4 Cambridge University Hospitals NHS Foundation Trust, Cambridge, United Kingdom; 5 Gynaecological Oncology Research Group, Division of Cancer Sciences, University of Manchester, Manchester, United Kingdom; 6 Manchester Academic Health Sciences Centre, Department of Obstetrics and Gynaecology, Manchester University NHS Foundation Trust, Manchester, United Kingdom

**Keywords:** Ovarian cancer, Predictive markers

## Abstract

**Background:**

Cancer antigen-125 (CA125) is widely used to investigate symptoms of possible ovarian cancer (OC) in primary care. However, OC risk varies with age and CA125 level. We externally validated the Ovatools models, which provide CA125- and age-specific OC risk.

**Methods:**

The performance of Ovatools in predicting OC diagnosis within 12 months of primary care CA125 was examined using English healthcare data for women <50 and ≥50 years. Discrimination and calibration were examined, accuracy was calculated at varying risk thresholds and compared to CA125 ≥ 35U/ml. We estimated OCs missed/detected by Ovatools in hypothetical diagnostic pathways, including a two-threshold pathway where moderate risk (1–2.9%) triggered primary care ultrasound, and higher risk (≥3%) triggered urgent cancer referral.

**Results:**

342,278 women were included, 0.63% had OC. The AUC was 0.95 in women ≥50 and 0.89 in women <50. When sensitivity/specificity were matched to CA125 ≥ 35U/ml, Ovatools showed marginally improved performance across other accuracy metrics in women ≥50 years. In a two-threshold pathway (≥50 years), 18.3% identified for urgent referral and 1% identified for ultrasound had OC.

**Discussion:**

Ovatools performed well on external validation. Ovatools could be used to support informed decision-making and to triage women for further investigation based on cancer risk.

## Introduction

Globally in 2022, there were ~324,000 new cases and 200,000 deaths from ovarian cancer (OC) [1]. In the United Kingdom (UK), 67% of women with OC are diagnosed with advanced disease, for which 5-year survival rates are 30% and 15% for stage III and IV, respectively [2]. Large trials have not demonstrated a mortality benefit from screening for OC [3, 4], and most women are diagnosed following a symptomatic presentation in primary care [5]. Cancer Antigen 125 (CA125) is used in many countries as the first-line test for possible OC in symptomatic women [6]. CA125 has reasonable accuracy to detect OC at the standard threshold (≥35U/ml) within English primary care with a Positive Predictive Value (PPV) for invasive OC of 9% [7]. However, the probability of OC varies markedly by both CA125 level and age, so older women with CA125 levels just below 35U/ml are more likely to have cancer than younger women with CA125 values well above this threshold [7]. For some tests, such as prostate-specific antigen (PSA), age-specific thresholds are employed in place of a single threshold, and this approach has been proposed for CA125 [8, 9].

The Ovatools prediction model was developed using CA125 results and age data from over 50,000 women tested in English primary care and provides the probability of OC to guide clinical decisions on the need for further investigation [10]. A National Institute for Health and Care Excellence (NICE) surveillance report recommended the national guidance on interpreting CA125 results in women with symptoms of possible OC is updated to incorporate age [8], informed by the development of the Ovatools modelling study [10]. A potential advantage of using risk models is that thresholds can be applied in line with national guidelines, such as the 3% risk threshold used in England for urgent cancer referral, thereby facilitating timely investigation in those at higher risk. This is of relevance to OC, as sequential primary care tests (CA125 followed by ultrasound) are required to trigger urgent cancer referral in England and several other countries, potentially contributing to prolonged periods of testing in primary care even in those at evidently higher risk. Evidence shows that the most common type of OC, high-grade serous, exhibits a median early stage (I-II) pre-diagnostic clinical phase of only 12 months [11], and that treatment delays of 1 month are associated with poorer survival [12], highlighting the need for accurate triage approaches and streamlined diagnostic pathways. More complex versions of the Ovatools models, incorporating additional variables (symptoms, ethnicity, body mass index, laboratory findings, breast cancer history) were developed previously but showed no improvement in the model’s diagnostic accuracy compared to using CA125 level and age only, so were not considered within the current study [10].

In this study, our primary aim was to externally validate the Ovatools models in a large representative primary care population, to assess model performance and generalisability. In addition, we sought to determine diagnostic accuracy to detect OC at clinically relevant risk thresholds and explore potential implications for OC detection when using different risk thresholds to guide further investigation or referral after a CA125 test within primary care in England.

## Methods

### Study design and data sources

This was a retrospective cohort study using English primary care data from the Clinical Research Practice Datalink (CPRD) Aurum dataset and linked cancer registry data from the National Cancer Registration and Analysis Service (NCRAS) [13, 14]. CPRD Aurum comprises anonymised, coded, electronic patient health records from GP surgeries using the EMIS clinical software [15] and is broadly representative of the UK population [16]. They include data on demographics, laboratory investigations, prescriptions, ethnicity and deprivation. NCRAS collects data on all patients in England diagnosed with cancer, including incidence date, histology, morphology, and stage at diagnosis. GP practices included in the model development study were excluded from the external validation dataset to ensure the sample was independent.

### Participants

We applied the same criteria used in the model development study [7] when defining the cohort but included data up to 2017 rather than up to 2014. We included women with a valid CA125 measurement recorded in CPRD between 1 May 2011 and 31 December 2017. The first CA125 test recorded during this period was the *index test*. Women <18 years on the index test date, those with a previous CA125 test in the year before their index test and those with a previous diagnosis of any OC (including borderline ovarian tumours) were excluded. Only CA125 values in standard units (U/ml, IU/ml, KU/L, KIU/ml) were included. CA125 entries were considered invalid if the value was missing, zero or below zero.

### Symptoms

In primary care in England, asymptomatic screening is not recommended and CA125 is only indicated in individuals with symptoms of possible OC [17]. We aimed to evaluate the overall performance of the Ovatools model in the real-world population of women tested using CA125. So, we included women with a primary care CA125 test regardless of the presence or absence of specific symptoms within their record. To examine common symptoms which may have triggered CA125 testing, we described the proportion of participants with codes indicating symptoms of possible OC [17] in the 90 days before index CA125 testing and performed sub-analyses examining model performance by the presence or absence of coded symptoms (Supplement 4.6).

### Clinical outcomes

The primary clinical outcome was invasive OC recorded in NCRAS within 12 months of the index CA125 test. Invasive OC was defined using the International Classification of Diseases (ICD)-10 codes by the World Health Organization, and included ovarian malignancy (C56), fallopian tube malignancy (C57.0), and primary peritoneal malignancy (C48.1, C48.2). Borderline ovarian tumours/neoplasms of uncertain behaviour of the ovary (D39.1) were excluded from the primary outcome. Given changes in the coding of borderline ovarian tumours over time, ICD-02 and ICD-03 tumour morphology and histology codes were reviewed in consultation with a clinical pathologist (BR) to ensure appropriate classification (Supplement 1). A sub-analysis was performed with early-stage (I-II) invasive OC as the outcome. We separately evaluated a second predictive model using any OC as the outcome, including borderline ovarian tumours and invasive OC in the outcome definition.

### Descriptive and demographic variables

Socioeconomic deprivation was measured at GP practice level using Townsend deprivation scores [18], and were grouped into quintiles, with quintile one being the least and five the most deprived. Ethnicity was categorised based on CPRD codes into five groups in line with the Office for National Statistics definitions: (i) Asian or Asian British, (ii) Black or Black British, (iii) Mixed, (iv) Other, and (v) White or White British [19]. Only the year of birth is recorded in CPRD to protect patient anonymity, therefore, a birthday and month of 1 July was assigned to all patients to derive age at index CA125 test.

### Statistical analysis

#### Estimating the risk of ovarian cancer

The Ovatools prediction models were originally developed using logistic regression and incorporated continuous CA125 level and continuous age, transformed using restricted cubic splines to account for non-linear relationships between variables. Separate models exist to predict the risk of (i) invasive OC and (ii) any OC, with full model specifications previously published [10]. For this study, we applied the prespecified models, using the same Knot placements for CA125 and age, to the external validation dataset. We used logistic regression to determine individuals’ log odds of invasive OC (Supplement 2), which were converted and reported as probability [0 to 1]. This was repeated for the any OC model. The hypothetical predicted risk of invasive OC and any OC that would occur for all ages 18 and 89 years (using age in years as a continuous variable) at all CA125 levels between 1 and 1000U/ml have been made available [20].

To simplify Ovatools use in practice, we also estimated mean predicted risks by CA125 level (1–1000U/ml) and age group (18–29 years, 30–39 years, 40–49 years, 50–59 years, 60–69 years, 70–79 years and 80–89 years) [21]. For example, two women aged 35 and 39 years with CA125 results of the same value would have the same predicted risk because they fall within the same age group. We report the closest integer CA125 values (U/ml) that equated to average Ovatools risks of ~1% and ~3% for each age group (Supplement 3) to demonstrate possible CA125 thresholds for ultrasound/urgent cancer referral by age group. These thresholds were chosen for examination in this study as ≥1% risk of cancer is often used by the NICE when recommending primary care tests in symptomatic patients, such as chest X-ray for possible lung cancer, and ≥3% as this threshold is used when recommending urgent cancer pathway referral [17].

#### External model validation

To assess Ovatools model performance using risk predictions by age (continuous) and risk predictions by age group, discrimination and calibration metrics were calculated. Discrimination is the ability to differentiate between those who experienced an event (invasive OC or any OC) from those who did not [22] and was determined by measuring the area under the curve (AUC). Calibration measures how closely predicted risk aligns with the proportion of those experiencing an outcome [23]. Mean calibration (calibration-in-the-large, CITL) and calibration slopes were provided when constructing calibration plots using the Stata package, *pmcalplot* [24, 25], and were used to calculate the intercept. Models with an intercept close to 0 and a slope close to 1 were considered well-calibrated. Good calibration is most important at risk levels close to potential clinical decision thresholds (1% and 3%). Therefore, we performed an additional analysis where participants with a predicted risk level >5% (1.7% of the cohort) were excluded from the calibration plot (Supplement 4.1). We also assessed for variation in the model’s performance using risk predictions (using continuous age and CA125) for the following demographics: (i) age (comparing women <50 and ≥50 years), (ii) ethnicity groups, and (iii) deprivation quintiles. The mean predicted risk was plotted against the mean outcome for each subgroup with demographic variable categories and performance metrics measured (AUC, slope, and intercept). We assessed the performance of (i) the invasive OC model (using continuous age) with early-stage invasive OC as the outcome (i.e. excluding missing stage, and stage III-IV), (ii) the any OC model (including borderline ovarian tumours), and (iii) the invasive OC model by presence/absence of coded OC symptoms (Supplement 4).

#### Diagnostic accuracy

Diagnostic accuracy was calculated using the Stata package, *diagt* [26], with the PPV, negative predictive value (NPV), sensitivity and specificity reported with 95% confidence intervals (CI) [27]. We calculated the diagnostic accuracy of Ovatools to predict invasive OC using (i) predicted risk by continuous age and (ii) age group, compared to using CA125 ≥ 35U/ml. We measured the accuracy of several example thresholds, including at ≥1% and ≥3% predicted risk, and compared this to CA125 ≥ 35U/ml. We also calculated the accuracy of using Ovatools risk levels with the same sensitivity as CA125 ≥ 35U/ml. The accuracy of Ovatools using risk predictions by continuous age was compared by demographic categories, (i) age (above and below 50 years), (ii) ethnicity and (iii) deprivation quintiles. We also report the diagnostic accuracy of using Ovatools-predicted risk by CA125 level and age group (Supplement 4.5).

#### Clinical implications

We estimated the number of women who had a CA125 test per year in England based on CPRD data and published population statistics [28, 29] (Supplement 5), and used the Ovatools accuracy metrics to approximate how many false/true positives and negatives would occur based on several exemplar pathways including one in which a 1–2.9% risk triggers primary care ultrasound and ≥3% risk triggers urgent cancer referral. This was calculated separately for women <50 and ≥50 years as well as when using risk predictions by age group. The health economic implications of the different thresholds and pathways were examined in a companion study [30]. All data management and analyses were conducted in Stata 18.0 [31].

### Sample size considerations

We calculated sample size requirements for precise estimation of observed divided by expected (O/E) cases, calibration slope, the C-statistic and net benefit at a referral threshold of 3%, using inputs from the development study and following guidance by Riley et al. [32], who recommend the sample size is at least as large as the maximum of the four required figures (Supplement 6). The largest of these values was 226,968 subjects.

### Patient and public involvement

Input was obtained from a patient and public involvement (PPI) group, some of whose members had experience with CA125 testing and OC. Preliminary study findings were shared and views on key findings and potential implications of the work for patients and the public were obtained and informed manuscript preparation. The PPI group will contribute to the dissemination of study findings.

## Results

### Participant characteristics

After applying the exclusion criteria, 342,278 participants were included in the study (Supplement 8). The median age was 53 years (interquartile range: 44–66). Most participants were categorised as White or White British (85%). Within 12 months of index CA125, 2143 (0.63%) women were diagnosed with invasive OC and 2655 (0.78%) with any OC (Table 1). Stage data was missing for 15% of women with invasive OC: of those with a recorded stage, 1247 (68%) had advanced disease (Stage III or IV). Most invasive OCs were of epithelial origin (92%) (Supplement 9).

**Table 1:** Cohort characteristics, cancer incidence, stage, and distribution of CA125 and Ovatools

		Participants, *n* (%)
Total		342,278
Age category in years	<30	18,319 (5.4)
	30–39	39,635 (11.6)
	40–49	85,344 (24.9)
	50–59	77,697 (22.7)
	60–69	57,257 (16.7)
	70–79	40,624 (11.9)
	≥80	23,402 (6.8)
Deprivation quintiles based on the Townsend Deprivation score [19]	Quintile 1 (least deprived)	83,628 (24.4)
	Quintile 2	75,611 (22.1)
	Quintile 3	66,688 (19.5)
	Quintile 4	57,796 (16.9)
	Quintile 5 (most deprived)	58,184 (17)
	Missing	371 (0.11)
Ethnicity	White or White British	289,186 (84.5)
	Asian or Asian British	17,711 (5.2)
	Mixed	14,261 (4.2)
	Black or Black British	11,290 (3.3)
	Other	5876 (1.7)
	Missing	3954 (1.2)
Distribution of CA125 and Ovatools	CA125 ≥ 35U/ml	23,742 (6.94)
	Ovatools risk 1–2.9%	18,342 (5.36)
	Ovatools risk ≥3%	9445 (2.76)
Cancer incidence	Invasive ovarian cancer	2143 (0.63)
	Borderline ovarian cancer	513 (0.15)
Stage at diagnosis (invasive ovarian cancer)	I	394 (18.4)
	II	186 (8.7)
	III	847 (39.5)
	IV	400 (18.7)
	missing	315 (14.7)

*CA125* cancer antigen 125, *n* number.

### Model external validation

Using predicted risk by continuous age, the invasive OC model showed good overall discrimination, with an AUC of 0.946 (95% CI: 0.939–0.953) (Table 2). The mean calibration was close to 0, the calibration slope was close to 1 and the intercept was close to 0, suggesting minimal overfitting. Model performance was tested by predicted risk level, and mean calibration was acceptable at thresholds of interest (~1% and ~3% risk) but there was less agreement between observed outcomes and predicted outcomes at extreme risk levels (Supplement 4.1). Performance was better for women ≥50 years (AUC 0.951, 95% CI: 0.942–0.960) than those <50 years (AUC 0.889, 95% CI: 0.886–0.892). The model performed well across deprivation levels and ethnicity groups, as well as between those with or without coded symptoms of possible OC (Supplement 4). When applying early-stage OC as the outcome, model performance was slightly lower (AUC 0.885, Intercept 0.0038, Slope 0.751). When using the any OC model including border tumours in the outcome, there was good calibration but marginally poorer discrimination (AUC 0.925, 95% CI: 0.918–0.931) than for the invasive OC model (Supplement 4.3). When using risk predictions by age group, the invasive OC model performed well, with only marginally lower metrics than when using risk predictions by continuous age (Supplement 4.4). Performance using age-group-based CA125 thresholds showed a similar pattern across ages compared to using the model with continuous age, with a better AUC for women ≥50 years (0.933, 95% CI: 0.922–0.943), compared to those <50 years (AUC 0.872, 95% CI: 0.842–0.901).

**Table 2:** Performance of the Ovatools invasive ovarian cancer model (using continuous age) on external validation

	Performance measurement (95% confidence interval)				
	Discrimination (AUC)	Calibration slope	Calibration intercept	CITL	O/E^a^
All ages	0.946 (0.939; 0.953)	1.004 (0.998; 1.010)	−0.0005 (−0.0007; −0.0003)	−0.102	0.931
Age <50 years	0.889 (0.886; 0.892)	1.063 (1.060; 1.066)	−0.0003 (−0.0005; −0.0001)	−0.065	0.942
Age ≥50 years	0.951 (0.942; 0.960)	0.998 (0.989; 1.007)	0.0000 (−0.0003; 0.0003)	−0.110	0.929

*AUC* area under the curve, *CA125* cancer antigen 125, *CITL* calibration in the large, *O/E* observed vs expected outcomes.

^a^Values below 1 indicate an over estimation of risk and values above 1 indicate an under estimation of risk.

### The diagnostic accuracy of Ovatools using continuous age vs CA125 ≥ 35U/ml

The diagnostic accuracy of Ovatools for invasive OC using continuous age is shown at risk thresholds ≥1% and ≥3% and compared to CA125 at ≥35 U/mL for women <50 and ≥50 years (Table 3). For women ≥50 years, Ovatools at ≥1% had a higher sensitivity than CA125 at ≥35U/ml (91.1% vs 86.5%) but lower PPV (7.2% vs 12.5%) and specificity (89.1% vs 94.3%). For women <50 years, Ovatools at ≥1% had a lower sensitivity than CA125 ≥ 35U/ml (61.9% vs 75.3%), with a greater specificity (96.9% vs 92.5%) and PPV (4.0% vs 2.0%). All diagnostic accuracy metrics for both Ovatools and CA125 ≥ 35U/ml were greater among women ≥50 than <50 years. When using the Ovatools risk thresholds with the same sensitivity and specificity as CA125 ≥ 35U/ml, Ovatools showed modest improvement across other accuracy metrics for women ≥50 years but not for women <50 years (Table 3). For women ≥50 years, an Ovatools threshold of ≥2.12% had the same sensitivity (86.5%) as CA125 at ≥35U/ml, but a greater specificity (94.8% vs 94.3%) and PPV (13.4% vs 12.5%). For women with early-stage invasive OC, Ovatools at ≥1% had a higher sensitivity than CA125 ≥ 35U/ml (70.7% vs 66.7%) but lower specificity (92.4% vs 93.6%) and a similar PPV (1.6% vs 1.7%) (Supplement 10).

**Table 3:** The diagnostic accuracy of Ovatools using predictions by continuous age at multiple risk thresholds, and CA125 at ≥35U/ml

Sub-group (invasive ovarian cancer incidence)	CA125 or Ovatools threshold	Sensitivity % (95% CI)	Specificity % (95% CI)	PPV % (95% CI)	NPV % (95% CI)
Age <50 years (0.21%)	CA125 ≥ 35 U/mL	75.3 (70.0; 80.0)	92.5 (92.3; 92.6)	2.0 (1.8; 2.3)	99.9 (99.9; 100)
	≥1%	61.9 (56.1; 67.4)	96.9 (96.8; 97.0)	4.0 (3.5; 4.7)	99.9 (99.9; 99.9)
	≥3%	45.2 (39.4; 51.0)	99.4 (99.3; 99.4)	12.7 (10.8; 14.9)	99.9 (99.9; 99.9)
	≥0.52%^a^	75.3 (70.0; 80.0)	91.6 (91.5; 91.7)	1.8 (1.6; 2.1)	99.9 (99.9; 100)
	≥0.57%^b^	73.6 (68.2; 78.5)	92.5 (92.4; 92.7)	2.0 (1.8; 2.3)	99.9 (99.9; 100)
Age ≥50 years (0.93%)	CA125 ≥ 35 U/mL	86.5 (84.8; 88.0)	94.3 (94.2; 94.4)	12.5 (11.9; 13.1)	99.9 (99.8; 99.9)
	≥1%	91.1 (89.7; 92.4)	89.1 (88.9; 89.2)	7.2 (6.9; 7.6)	99.9 (99.9; 99.9)
	≥3%	83.1 (81.3; 84.8)	96.5 (96.4; 96.6)	18.3 (17.5; 19.1)	99.8 (99.8; 99.9)
	≥2.12%^c^	86.5 (84.8; 88.0)	94.8 (94.7; 94.9)	13.4 (12.8; 14.1)	99.9 (99.8; 99.9)
	≥1.95%^d^	87.7 (86.1; 89.1)	94.3 (94.2; 94.4)	12.5 (11.9; 13.1)	99.9 (99.9; 99.9)

*CA125* cancer antigen 125, *CI* confidence interval, *NPV* negative predictive value, *PPV* positive predictive value.

The following Ovatools thresholds were selected because they had the same sensitivity^a,c^ or specificity^b,d^ as CA125 ≥ 35U/ml.

### CA125 levels equating to Ovatools risk ~1% and ~3% by age group

**Fig. 1:**
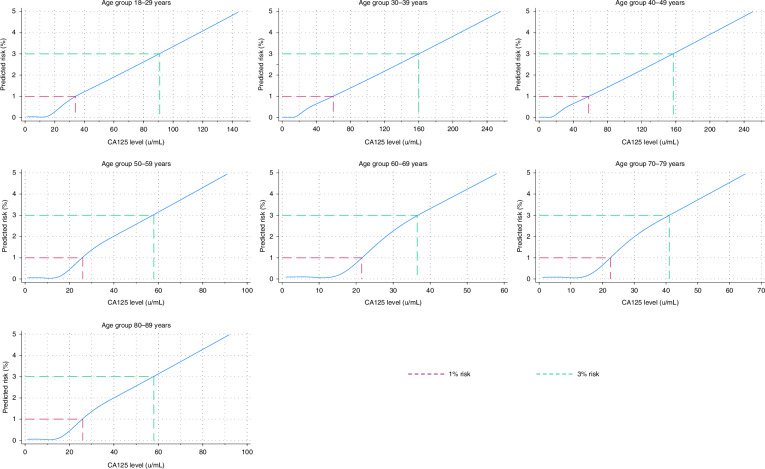
The predicted risk of invasive ovarian cancer by CA125 level and age group using Ovatools, and CA125 levels equating to ~1% and ~3% risk.

The CA125 levels equating to Ovatools risk levels of ~1% and ~3% for invasive OC by age group are displayed in Fig. 1 (numerical values are in Supplement 3). CA125 values were highest in women aged 30–39 years (1%: CA125 = 59U/ml, 3%: CA125 = 160U/ml) and 40–49 years (1%: CA125 = 58U/ml, 3%: CA125 = 157U/ml), and lowest in women in the 60–69 years age group (1%: CA125 = 22U/ml, 3%: CA125 = 37U/ml). The CA125 level equating to ~1% Ovatools risk was higher than the current CA125 threshold (≥35U/ml) in age groups 30–39 years and 40–49 years but lower in all other age groups. In the 60–69 years group, the CA125 value equating to ~3% risk was only 2U/ml higher than the current CA125 threshold for primary care ultrasound. Ovatools thresholds equating to ~1% risk had a greater sensitivity for all age groups except for women 30–39 years and 40–49 years, when compared to CA125 at ≥35U/ml (Supplement 4.5).

### Clinical implications of using Ovatools in general practice

**Fig. 2:**
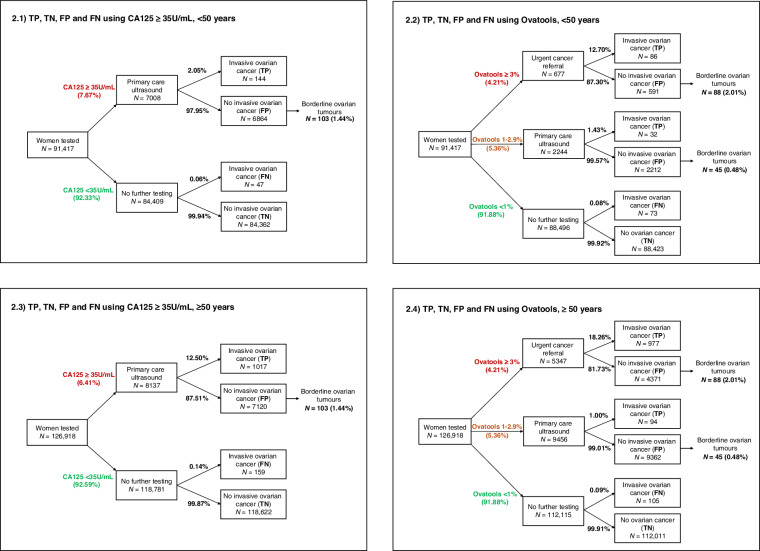
Estimated true positive (TP), false positive (FP), false negative (FN) and true negative (TN) cases that could occur in England per year using the CA125 ≥ 35U/ml to trigger primary care ultrasound compared to using Ovatools at 1–2.9% risk to trigger primary care ultrasound and ≥3% to trigger urgent cancer referral for women <50 years (2.1 & 2.2), and women ≥50 years (2.3 & 2.4). Fig. 2.1. TP, TN, FP and FN using CA125 ≥ 35U/ml, <50 years. Fig. 2.2. TP, TN, FP and FN using Ovatools, <50 years. Fig. 2.3. TP, TN, FP and FN using CA125 ≥ 35U/ml, ≥50 years. Fig. 2.4. TP, TN, FP and FN using Ovatools, ≥50 years.

In England in 2022, we estimated 218,335 women over 18 years were tested using CA125 in primary care, with 57.7% (*n* = 126,918) of the population being ≥50 years and 42.3% (*n* = 91,417) being <50 years. Using standard practice, 8137 (6.4%) of CA125-tested women ≥50 years and 7008 (7.7%) <50 years would have a CA125 result ≥35U/ml and therefore qualify for primary care ultrasound under NICE guidance (Fig. 2 and Table 4). By comparison, if an Ovatools risk of 1–2.9% triggered primary care ultrasound and ≥3% triggered urgent cancer pathway referral, 14,803 (11.7%) and 2921 (3.2%) of women ≥50 years and <50 years, respectively, would qualify for further investigation following CA125 (ultrasound or urgent cancer referral). This equates to 6666 more women ≥50 years but 4087 fewer women <50 years being investigated further following CA125 testing (Table 4). This approach would result in 54 additional women with OC ≥ 50 years but 26 fewer women with OC < 50 years being identified for investigation in England per year. If the Ovatools pathway at the proposed thresholds were used for women ≥50 years only, 1 in 5 (18.3%) high-risk women (≥3% Ovatools risk) identified for urgent cancer referral, and 1 in 100 moderate-risk women (1–2.9% Ovatools risk) identified for primary care ultrasound would have invasive OC. For every 123 additional women ≥50 years sent for further investigation using Ovatools compared to standard practice, 1 additional case of invasive OC could be identified.

**Table 4:** Number of women sent for further testing and detected with invasive OC using CA125 ≥ 35U/ml and Ovatools at multiple thresholds (using continuous age).

Age group	Threshold for primary care ultrasound	USS following CA125	Change in pelvic ultrasounds using Ovatools	Urgent cancer referrals (risk ≥3%)	Additional women testing further using Ovatools	Invasive OC detected	Additional OC detected using Ovatools	Additional OC diagnoses/100,000 CA125-tested women
≥50 years	CA125 ≥ 35 U/mL	8137	NA	NA	NA	1017	NA	NA
	Ovatools 1–2.9%	9456	1319	5347	6666	1071	54	59
	Ovatools^a^ 1.95–2.9%	2906	−5231	5347	116	1031	14	15
	Ovatools^b^ 2.12–2.9%	2222	−5915	5347	−568	1017	0	0
<50 years	CA125 ≥ 35 U/mL	7008	NA	NA	NA	144	NA	NA
	Ovatools 1–2.9%	2244	−4764	678	−4086	118	-26	-28

*CA125* cancer antigen 125, *OC* ovarian cancer.

Ovatools threshold with a matched specificity^a^ and sensitivity^b^ to CA125 > 35U/ml for women ≥50 years.

Table 4 demonstrates the clinical utility of using Ovatools risk thresholds with matched sensitivity (risk ≥2.12%) and specificity (risk ≥1.95%) to that of CA125 ≥ 35U/ml for women ≥50 years. For women ≥50 years, if 2.12–2.9% risk triggered primary care ultrasound, while ≥3% triggered urgent cancer referral, 568 fewer women would be sent for further investigation compared to standard practice while identifying the same number of invasive OC cases. If 1.95–2.9% risk triggered primary care ultrasound and ≥3% triggered urgent cancer referral, only 116 additional women would be investigated further while identifying 14 additional cases of invasive OC (1 in 8 women tested further). Clinical utility was repeated using age-based CA125 thresholds approximating ~1% and ~3% risk to trigger ultrasound or referral, respectively, showing similar findings, with an increase in OC cases detected in women ≥50 years, but additional missed cases in women <50 years (Supplement 5).

## Discussion

This study found that Ovatools, an age- and CA125-based model to predict the risk of invasive OC, performed well on external validation in 342,278 CA125-tested women in English primary care. Performance was similar across ethnic and sociodemographic groups but greater in women ≥50 than <50 years. Depending on the threshold(s) chosen, Ovatools age-adjusted risk thresholds reduced false positives or false negatives when compared to CA125 ≥ 35U/ml. When the sensitivity or specificity of Ovatools thresholds were matched to CA125 ≥ 35U/ml, Ovatools exhibited a modest improvement across other accuracy metrics (PPV, NPV) for women ≥50 years and a reduction in primary care ultrasounds among women without OC. In a scenario where moderate Ovatools risk (1–2.9%) triggers primary care ultrasound and higher Ovatools risk (≥3%) triggers urgent cancer referral for women ≥50 years, more women would be selected for investigation in comparison to the current practice, with 1 in 123 additional women selected for further testing having invasive OC, and 1 in 5 (18.3%) high-risk women selected for direct urgent cancer referral having invasive OC. In women <50 years, the same risk thresholds would result in fewer unnecessary referrals (false positives) but additional missed OC cases (false negatives) when compared to current practice due to the ≥1% risk threshold having a lower sensitivity than current practice, so alternative thresholds or diagnostic strategies may be more appropriate for this age group and warrants further investigation.

The Ovatools model enables thresholds for further investigation to be chosen based on age- and CA125-derived OC risk. Model parameters and risk levels equating to a wide range of CA125 levels and ages are published alongside this paper to enable thresholds to be set in line with local or national priorities. Results could also be used to inform individual doctor-patient choices on further investigation. Using an Ovatools threshold (>2.12% risk) with an equivalent sensitivity to CA125 ≥ 35U/ml for women ≥50 years could reduce unnecessary ultrasound investigations without increasing missed OC cases. However, to achieve earlier detection, more sensitive methods to identify symptomatic OC in primary care and fast-track high-risk women through the diagnostic pathway are needed, particularly in the absence of OC screening [3]. Ovatools could be used to select high-risk women for expedited investigation, such as ≥3% risk to trigger urgent cancer referral in CA125-tested women in line with the 3% NICE threshold for urgent referral of symptomatic patients for other cancers [17]. This approach would enable expedited specialist gynaecological assessment and ultrasound using the gold standard Risk of Malignancy Index or IOTA for women with a high risk of undiagnosed invasive OC [33]. Under current NICE guidelines, these high-risk women require a GP-requested ultrasound before urgent referral, which can take weeks or months [34], are associated with longer primary care intervals in OC [35] and are generally not interpreted using gold standard approaches. The proportion of women in the ≥3% risk Ovatools group who had invasive OC (12.7% and 18.3% of women <50 and ≥50 years, respectively) would far exceed the current gynaecological urgent cancer referral pathway conversion rate for England (2.9%) [36]. In addition, offering women at low-but-not-no-risk (1–2.9%) of invasive OC ultrasound in primary care (the current standard in women with raised CA125) could reduce false negative results, which are associated with longer test-diagnostic intervals [37]. Such approaches have the potential to increase timely OC detection but would rely on improved timely access to transvaginal ultrasound within primary care, which is currently planned in England as part of the expansion of community diagnostic centres [38].

Applying age-specific thresholds would have significant implications for patients and the healthcare service. In a companion study, we found that using a two-threshold pathway where 1-2.9% and ≥3% risk triggers ultrasound and urgent referral respectively, is likely to be cost-effective for women ≥50 years when compared to the current NICE-recommended practice [30]. However, before further implementation is considered, feasibility and acceptability of any changes should also be explored. Further, while CA125 is used as the first-line test in England and several other countries, elsewhere CA125 and ultrasound (or computed tomography) are used in parallel and different Ovatools risk thresholds may be more appropriate for those settings [6]. While Ovatools risk at individual ages and CA125 levels could be readily incorporated into blood test reports to inform individual decision-making, we evaluated the CA125 levels equating to clinically relevant thresholds by age group as these may be easier to implement. Similar approaches have been used for other tests such as PSA, with NICE guidelines recently updated to recommend referral at different PSA levels in different age groups [8].

Few studies have examined the performance of CA125 or OC prediction models in primary care in the UK. In the current study, CA125 (≥35U/ml) had the same sensitivity and specificity to detect invasive OC as at model development; however, we demonstrated a slightly lower PPV (7.7% vs 8.8%), and lower incidence of any OC (0.78% vs 0.93%). As found in the development study [10], the Ovatools model performed less well in women <50 years. This is likely due to CA125 itself performing less well in younger women [7] (in part due to differences in tumour morphology [39]), the lower incidence of OC in this group, and greater incidence of benign conditions which can elevate CA125. We found that a high proportion (42%) of CA125 tests were performed in women <50, but the incidence of OC was five times lower than in the group ≥50 years, while only 2% of women <50 years with a CA125 ≥ 35U/ml had an OC. Given the limited accuracy of CA125 and Ovatools in younger women, other tests could be considered, such as HE4, a biomarker shown to have high sensitivity to detect OC when used alongside CA125 in symptomatic women <50 years [40, 41]. In a separate study, we modelled the harm/cost and benefit of using Ovatools in the English primary care setting and found that is was potentially cost effective for women over 50 years, where ≥1% and ≥3% risk triggers ultrasound or urgent cancer referral, respectively [30]. However, future research needs to be conducted to determine the feasibility of using Ovatools in English primary care settings, including evaluating how the model could best be integrated into clinical practice and workflows. For example, a woman’s risk could be provided alongside the CA125 result within laboratory reports with a clinical recommendation for action, or age-adjusted CA125 thresholds could be provided to aid interpretation, similar to those currently used to interpret PSA results in UK general practice. Further research is planned to examine options.

### Strengths and limitations

This study used a large dataset which was broadly representative of the English population, with ethnicity and deprivation distributions similar to national estimates [42, 43]. Laboratory results are automatically recorded in CPRD, leading to high levels of completeness. We did not have access to data on CA125 assay type and so could not account for assays variability within this study. While assay variability is also an issue within current practice, with a single CA125 threshold recommended regardless of the assay used, we advocate further research to consider the impact of assay variably and calibration between assays and labs to support implementation of Ovatools models [44]. We were unable to determine the reasons for CA125 testing due to the use of routine data. However, our results are based on CA125 tests done on women within English primary care and therefore provide an indication of model performance in real-world clinical practice, irrespective of individual’s clinical presentation. We did not include symptoms or demographic variables other than age in the model as they were previously shown to have no effect on performance [10]. Furthermore, complex prediction models are more challenging to implement, and few are routinely used in primary care. However, we did examine variation in performance by key demographic factors (age group, ethnicity and deprivation) and the presence of coded symptoms, showing that the models performed well across all groups. The completeness and accuracy of NCRAS data used to determine outcomes are high [45]. However, stage data was missing for 15% of invasive OCs, limiting analysis by stage. Not all women had a reference standard test and instead we rely on OC diagnoses within 12 months of CA125. Some women may be diagnosed beyond this interval, and some may develop cancer during the period following CA125 testing which may introduce bias [46, 47]. A 12- month period has been widely used in similar research [7, 37] and was chosen as a compromise between minimising the inclusion of new cancers and maximizing the inclusion of relevant cancers.

## Conclusion

Ovatools performs well in identifying invasive OC in CA125-tested women, particularly in women ≥50 years. The model could be used to interpret CA125 levels within primary care and select higher-risk women for further investigation and referral. This approach has the potential to expedite diagnosis, but further work is needed to understand the feasibility and acceptability of using Ovatools within diagnostic pathways.

## Supplementary information

Supplementary files 1-10

Dataset containing the predicted risk by age category and CA125 level

Dataset containing the predicted risk by age (continuous) and CA125 level

## Data Availability

The data used for this study were provided by CPRD and NCRAS and are subject to a licensing agreement that prohibits sharing outside the research team. Data can be requested through CPRD. All Stata scripts and code lists used to clean and analyse the data will be made available on the Queen Mary Research Online, an online data repository, or as supplementary materials.
