# Supplementary material for: CA125 and age-based models for ovarian cancer detection in primary care: a population-based external validation study
Source: Br J Cancer. 2025 Sep 17;133(10):1483–91. doi: 10.1038/s41416-025-03165-4 (PMC12603205; doi:10.1038/s41416-025-03165-4)
Supplement: Supplementary file 1 — Supplementary files 1-10 [file 41416_2025_3165_MOESM1_ESM.pdf]

Supplement 1: International Classification of Disease (ICD)-02 / 03 morphological classification of ovarian tumours

morphology code	behaviour	category	Description (term)
80001	borderline	unknown	neoplasm, uncertain whether benign or malignant
80011	borderline	unknown	tumour cells uncertain whether benign or malignant
80101	borderline	epithelial (other)	epithelial tumour, uncertain behaviour
80105	borderline	epithelial (unknown)	carcinoma in situ with microinvasion
81405	borderline	epithelial (unknown)	adenoma
82401	borderline	non-epithelial	carcinoid tumour nos
83131	borderline	epithelial (clear cell)	clear cell adenofibroma of borderline malignancy
83801	borderline	epithelial (endometrioid)	endometrioid adenoma, borderline malignancy
83811	borderline	epithelial (endometrioid)	endometrioid adenofibroma borderline malignancy
84401	borderline	epithelial (unknown)	cystadenocarcinoma borderline malignancy
84411	borderline	epithelial (serous)	serous cystadenoma, borderline malignancy
84415	borderline	epithelial (serous)	serous cystadenoma nos
84421	borderline	epithelial (serous)	serous borderline tumor, nos
84422	borderline	epithelial (serous)	borderline serous tumour with high grade dysplasia
84423	borderline	epithelial (serous)	serous cystadenoma, borderline malignancy
84425	borderline	epithelial (serous)	serous cystadenoma, microinvasion
84441	borderline	epithelial (clear cell)	clear cell cystic tumor of borderline malignancy
84511	borderline	epithelial (unknown)	papillary cystadenoma, borderline malignancy
84513	borderline	epithelial (unknown)	papillary cystadenoma borderline malignancy
84515	borderline	epithelial (unknown)	papillary cystadenoma, borderline malignancy
84601	borderline	epithelial (serous)	papillary serous cystadenoma borderline malignancy
84605	borderline	epithelial (serous)	papillary serous cystadenoma, nos
84611	borderline	epithelial (serous)	serous surface papilloma borderline malignancy
84621	borderline	epithelial (serous)	serous papillary cystic tumor of borderline malignancy
84623	borderline	epithelial (serous)	papillary serous cystadenoma, borderline malignancy
84625	borderline	epithelial (serous)	papillary serous cystadenoa, microinvasion
84631	borderline	epithelial (serous)	serous surface papillary tumor of borderline malignancy
84701	borderline	epithelial (mucinous)	cystadenoma mucinous b/l
84705	borderline	epithelial (mucinous)	mucinous cystadenoma
84721	borderline	epithelial (mucinous)	mucinous cystic tumor of borderline malignancy
84722	borderline	epithelial (mucinous)	mucinous cystic tumor of borderline malignancy with intraepithelial carcinoma
84723	borderline	epithelial (mucinous)	mucinous cystadenoma, borderline malignancy
84725	borderline	epithelial (mucinous)	mucinous cystadenoma, microinvasive
84731	borderline	epithelial (mucinous)	papillary mucinous cystadenoma borderline malignan
84733	borderline	epithelial (mucinous)	papillary mucinous cystadenoma, borderline malignancy
84741	borderline	epithelial (other)	seromucinous borderline tumour
84801	borderline	epithelial (mucinous)	low grade appendiceal mucinous neoplasm
84811	borderline	epithelial (mucinous)	mucin-producing adenocarcinoma borderline malignan
85901	borderline	non-epithelial (sex cord stromal)	sex cord stromal tumour
86211	borderline	non-epithelial (sex cord stromal)	granulosa cell theca-cell tum
86221	borderline	non-epithelial (sex cord stromal)	granulosa cell tumor, juvenile (not testis)
86311	borderline	non-epithelial (sex cord stromal)	sertoli-leydig cell tumor of intermediate differentiation
86321	borderline	non-epithelial (sex cord stromal)	gynandroblastoma
86401	borderline	non-epithelial (sex cord stromal)	sertoli cell tumour
86501	borderline	non-epithelial (sex cord stromal)	leydig cell tumour nos
88101	borderline	non-epithelial (sex cord stromal)	cellular fibroma

88971	borderline	non-epithelial	smooth muscle tumour of uncertain malignant potent
89351	borderline	non-epithelial (sex cord stromal)	stromal tumor, nos
89901	borderline	non-epithelial	mesenchymoma, nos
90001	borderline	epithelial (other)	brenner tumor, borderline malignancy
90003	borderline	epithelial (other)	brenner tumor, malignant
90131	borderline	epithelial (unknown)	adenofibroma borderline malignancy
90141	borderline	epithelial (serous)	serous adenofibroma of borderline malignancy
90151	borderline	epithelial (mucinous)	mucinous adenofibroma of borderline malignancy
90731	borderline	non-epithelial (germ cell)	gonadoblastoma
90801	borderline	non-epithelial (germ cell)	teratoma
90911	borderline	non-epithelial (germ cell)	strumal carcinoid
91101	borderline	epithelial (other)	mesonephric tumour, nos
80003	invasive	unknown	neoplasm, malignant
80009	invasive	epithelial (other)	neoplasm, malignant unknown if primary or metastatic
80043	invasive	epithelial (unknown)	malignant tumor, spindle cell type
80053	invasive	epithelial (clear cell)	malignant tumor, clear cell type
80103	invasive	epithelial (unknown)	carcinoma nos
80133	invasive	epithelial (other)	large cell neuroendocrine carcinoma
80203	invasive	epithelial (other)	carcinoma, undifferentiated nos
80213	invasive	epithelial (unknown)	carcinoma anaplastic type
80223	invasive	epithelial (unknown)	pleomorphic carcinoma
80333	invasive	epithelial (other)	pseudosarcomatous carcinoma
80413	invasive	epithelial (other)	small cell carcinoma nos
80443	invasive	epithelial (other)	small cell carcinoma, hypercalcaemic type
80463	invasive	epithelial (unknown)	non-small cell carcinoma
80503	invasive	epithelial (unknown)	papillary carcinoma nos
80523	invasive	epithelial (other)	papillary squamous cell carcinoma
80703	invasive	epithelial (other)	squamous cell carcinoma nos
80713	invasive	epithelial (other)	squamous cell carcinoma, keratinizing, nos
80733	invasive	epithelial (other)	squamous cell carcinoma small cell non-keratinisin
81203	invasive	epithelial (other)	transitional cell ca nos
81403	invasive	epithelial (unknown)	adenocarcinoma nos
81409	invasive	epithelial (unknown)	adenocarcinoma, nos unknown if primary or metastatic
81443	invasive	epithelial (unknown)	adenocarcinoma intestinal type
82403	invasive	non-epithelial	carcinoid tumor nos
82433	invasive	non-epithelial	goblet cell carcinoid
82463	invasive	epithelial (other)	neuroendocrine carcinoma
82493	invasive	non-epithelial	atypical carcinoid/grade 2 neuroendocrine tumour
82553	invasive	epithelial (unknown)	adenocarcinoma with mixed subtypes
82603	invasive	epithelial (unknown)	adenocarcinoma papillary nos
83103	invasive	epithelial (clear cell)	clear cell adenocarcinoma nos
83203	invasive	non-epithelial (sex cord stromal)	granular cell carcinoma
83233	invasive	epithelial (other)	mixed cell adenocarcinoma
83803	invasive	epithelial (endometrioid)	endometrioid carcinoma
83823	invasive	epithelial (endometrioid)	endometrioid adenocarcinoma, secretory variant
84403	invasive	epithelial (unknown)	cystadenocarcinoma nos
84413	invasive	epithelial (serous)	serous cystadenocarcinoma nos
84503	invasive	epithelial (unknown)	cystadenocarcin papillary nos
84603	invasive	epithelial (serous)	papillary serous cystadenocarcinoma
84613	invasive	epithelial (serous)	serous surface papillary carcinoma
84703	invasive	epithelial (mucinous)	mucinous cystadenocarcinoma nos
84713	invasive	epithelial (mucinous)	papillary mucinous cystadenocarcinoma
84743	invasive	epithelial (other)	seromucinous carcinoma
84803	invasive	epithelial (mucinous)	mucinous adenocarcinoma
84813	invasive	epithelial (mucinous)	mucin-producing adenocarcinoma

84823	invasive	epithelial (mucinous)	mucinous adenocarcinoma, endocervical type
84903	invasive	epithelial (mucinous)	signet ring cell carcinoma
85603	invasive	epithelial (other)	adenosquamous carcinoma
85743	invasive	epithelial (unknown)	adenocarcinoma with neuroendocrine differentiation
85753	invasive	epithelial (unknown)	metaplastic carcinoma, nos
86003	invasive	non-epithelial (sex cord stromal)	theca cell carcinoma
86201	invasive	non-epithelial (sex cord stromal)	granulosa cell tumor nos
86203	invasive	non-epithelial (sex cord stromal)	granulosa cell tumor, malignant
86313	invasive	non-epithelial (sex cord stromal)	sertoli-leydig cell tumour, poorly differentiated
86503	invasive	non-epithelial (sex cord stromal)	leydig cell tumor, malignant
86703	invasive	non-epithelial	steroid cell tumor, malignant
88003	invasive	non-epithelial	sarcoma nos
88103	invasive	non-epithelial	fibrosarcoma
88513	invasive	non-epithelial	liposarcoma, well differentiated
88903	invasive	non-epithelial	leiomyosarcoma nos
89303	invasive	non-epithelial	endometrial stromal sarcoma, nos
89313	invasive	non-epithelial	endometrial stromal sarcoma, low grade
89333	invasive	non-epithelial	adenosarcoma
89363	invasive	non-epithelial	gastrointestinal stromal sarcoma
89403	invasive	unknown	mixed tumour malignant
89503	invasive	epithelial (other)	mullerian mixed tumor
89513	invasive	epithelial (other)	mesodermal mixed tumor
89803	invasive	epithelial (other)	carcinosarcoma, nos
89903	invasive	non-epithelial	mesenchymoma malignant
90143	invasive	epithelial (serous)	serous adenocarcinofibroma
90603	invasive	non-epithelial (germ cell)	dysgerminoma
90643	invasive	non-epithelial (germ cell)	germinoma
90713	invasive	non-epithelial (germ cell)	endodermal sinus tumour
90803	invasive	non-epithelial (germ cell)	teratoma, malignant nos
90813	invasive	non-epithelial (germ cell)	teratocarcinoma
90833	invasive	non-epithelial (germ cell)	malignant teratoma, intermediate
90843	invasive	non-epithelial (germ cell)	dermoid cyst with mal transform
90853	invasive	non-epithelial (germ cell)	mixed germ cell tumor
90903	invasive	non-epithelial (germ cell)	struma ovarii, malignant
91103	invasive	epithelial (other)	mesonephroma malignant
93643	invasive	other	ewing sarcoma / pnet
95003	invasive	other	neuroblastoma

```
****SUPPLEMENT 2

****This code was applied using Stata version 18.


/*

CALCULATE THE RISK OF INVASIVE OVARIAN & OVARIAN FOR EACH PARTICIPANT BASED ON
THE  PREDICTIVE MODEL FORMULA USING CA125 AND AGE


ORIGINAL KNOTS:

Log ca125                  -1.390562 -0.8027754 -0.5150933 -0.1667867 0.7376696

Age mean centred           -24 -9 -1 10 27


*/

cd "G:\Primary Care\OVATOOLS\Data\Data prep"

use cohort, clear

keep epatid CA125 test age ovaryca invasive age_cat


***create new splines for new dataset:

*log transform CA125, floor & mc

gen log_ca125=log(CA125)

sum log_ca125, d // mean 2.569051

*replace log_ca125=log_ca125-2.569051

replace log_ca125=log_ca125-3

sum log_ca125, d


*mean centre and floor age

sum age, d // mean 54.71233

replace age=floor(age)

gen agemc=age-55

order epatid CA125 age agemc

sum agemc, d

sort age CA125
```

```
*gen splines for new dataset

mkspline log_ca125_=log_ca125, cubic knots (-1.390562 -0.8027754 -0.5150933 -0.1667867 0.7376696)

matrix K=r(knots)


mkspline age_=agemc, cubic knots (-24 -9 -1 10 27)

matrix KA=r(knots)


********************************************************************************

********************************************************************************

*CALCUATE INDIVIDUAL RISK FOR EACH MODEL

*OVARIAN CANCER MODEL

gen log_odds_ovary=ln(0.0002362) +(log_ca125_1*ln(2.26625)) +(log_ca125_2*ln(0.0046567)) +(log_ca125_3*ln(1.25*10^31)) +(log_ca125_4*ln(3.40*10^-56)) + (age_1*ln(0.9263145)) + (age_2*ln(1.746288)) + (age_3*ln(0.1047997)) + (age_4*ln(7.06957))


*INVASIVE-OVARIAN CANCER MODEL

gen log_odds_invasive=ln(0.0000571) +(log_ca125_1*ln(1.069068)) +(log_ca125_2*ln(0.0007832)) +(log_ca125_3*ln(8.33*10^43)) +(log_ca125_4*ln(3.36*10^-79)) + (age_1*ln(0.9415075)) + (age_2*ln(1.829243)) + (age_3*ln(0.0776767)) + (age_4*ln(10.23064))


*convert log odds to probability [0,1]

gen risk_ovary=exp(log_odds_ovary)/(1+exp(log_odds_ovary))


*invasive ovarian cancer risk

gen risk_invasive=exp(log_odds_invasive)/(1+exp(log_odds_invasive))


save risk_all_models, replace
```

Supplement 3: Average CA125 levels equating to approximately 1-2.9% and ≥3% risk of invasive ovarian cancer by age group using the Ovatools model

Age group	Equivalent CA125 level (U/ml)	
	Ovatools 1-2.9% risk	Ovatools ≥3% risk
18-29 years	34-90.9	≥91
30-39 years	59-159.9	≥160
40-49 years	58-156.9	≥157
50-59 years	26-56.9	≥57
60-69 years	22-36.9	≥37
70-79 years	22-40.9	≥41
80-89 years	26-57.9	≥58

Supplement 4: Additional model validation metrics

4.1. Performance by risk level

Participants were ranked by ascending predicted risk level and grouped into bins of 5000. The mean predicted risk was plotted against the mean outcome for each bin (each bin is represented by a small blue dot in the graph). Predicted risk levels above 5% are not displayed because they were considered outliers, are not clinically relevant and formed a very small proportion of the population sample (1.18%). For lower risk predictions, the model slightly under predicted risk, and for higher predictions the model overpredicted risk. Agreement between predicted and observed outcomes was greater for lower prediction levels.

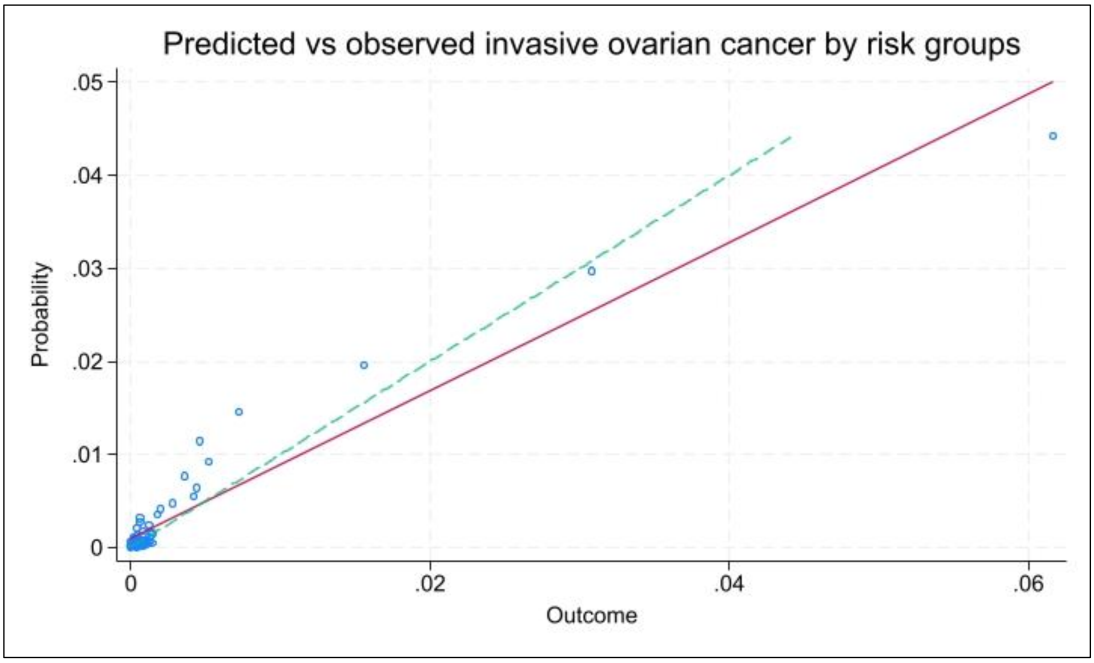

4.2. Invasive ovarian cancer model performance by ethnicity, deprivation and early-stage invasive ovarian cancer

		AUC	Intercept	Slope	O:E	CITL
Early-stage invasive OC (excludes missing & stage III-IV)		0.885 (0.867-0.902)	0.0038 (0.0037-0.0040)	0.751 (0.751-0.751)	0.333	-1.344
Ethnicity group	Asian/Asian British	0.899 (0.849-0.950)	0.0007(0.0000-0.0015)	0.900 (0.899-0.901)	0.896	-0.140
	Black/Black British	0.953 (0.900-1.000)	0.0015 (0.0009-0.0022)	1.119 (1.118-1.120)	0.554	-0.745
	Mixed	0.944 (0.894-0.993)	0.0017 (0.0011-0.0024)	0.989 (0.988-0.990)	0.603	-0.675
	White/White British	0.947 (0.940-0.954)	0.0003 (0.0001-0.0005)	1.004 (1.004-1.004)	0.958	-0.063
	Other	0.936 (0.857-1.000)	0.0003 (-0.0008-0.0014)	1.114 (1.113-1.115)	0.827	-0.239
Deprivation quintiles (1=least, 5=most deprived)	Quintile 1	0.947(0.934-0.960)	0.0003 ((-0.0001)-0.0007)	1.008 (1.008-1.008)	0.952	-0.073
	Quintile 2	0.953 (0.941-0.965)	0.0003 ((-0.0001)-0.0008)	1.005 (1.005-1.005)	0.948	-0.078
	Quintile 3	0.940 (0.926-0.955)	0.0004 ((-0.0001)-0.0008)	0.977 (0.977-0.977)	0.972	-0.041
	Quintile 4	0.942 (0.925-0.958)	0.0003 ((-0.0002)-0.0007)	1.005 (1.005-1.005)	0.952	-0.069
	Quintile 5	0.945 (0.926-0.965)	0.0011 (0.0007-0.0015)	1.030 (1.030-1.030)	0.775	-0.334
AUC=area under the curve; CITL = calibration in the large; OC = ovarian cancer; O:E=observed vs expected outcomes						

4.3. Any ovarian cancer model performance for all participants

AUC (95% CI)	Intercept (95% CI)	Slope (95% CI)	O:E	CITL
0.925 (0.918-0.931)	0.0009 (0.0007-0.0011)	1.010 (1.010-1.010)	0.889	-0.159
AUC = area under the curve, CITL = calibration-in-the-large, O:E = observed versus expected				

4.4. Invasive ovarian cancer model performance for all participants using age group and CA125 level

All group	Performance measurement (95% confidence interval)				
	AUC	Calibration slope	Calibration intercept	CITL	O:E
All ages, 18-89 years	0.947 (0.940-0.954)	1.001 (0.994-1.008)	0.0015 ((-0.0054)-0.0083)	-0.111	0.926
All ≥50 years	0.951 (0.943-0.958)	0.992 (0.983-1.001)	0.0098 (0.0005-0.0192)	-0.119	0.925
All <50 years	0.897 (0.873-0.922)	1.073 (1.070-1.076)	0.0022 ((-0.0009)-0.0052)	-0.077	0.932
18-29 years	0.804 (0.689-0.920)	0.789 (0.787-0.791)	0.0015 ((-0.0009)-0.0038)	-0.683	0.520
30-39 years	0.885 (0.830-0.941)	0.942 (0.940- 0.944)	0.0014 ((-0.0011)-0.0039)	-0.319	0.738
40-49 years	0.912 (0.885-0.939)	1.144 (1.141-1.147)	0.0026 ((-0.0008)-0.0061)	0.118	1.110
50-59 years	0.922 (0.900-0.943)	1.042 (1.036-1.048)	0.0056 ((-0.0005)-0.0116)	-0.076	0.945
60-69 years	0.967 (0.957-0.976)	1.000 (0.990-1.010)	0.0112 (0.0013-0.0211)	-0.140	0.918
70-79 years	0.959 (0.948-0.969)	0.970 (0.957-0.983)	0.0141 (0.0015-0.0267)	-0.112	0.932
80-89 years	0.936 (0.915-0.957)	0.931 (0.917-0.945)	0.0138 (0.0001-0.0276)	-0.158	0.899
AUC=area under the curve; CITL = calibration in the large; O:E=observed vs expected outcomes					

4.5. The diagnostic accuracy of using CA125 thresholds equating to ~1% and ~3% Ovatools risk by age group compared to CA125 ≥35U/ml to detect invasive ovarian cancer

Age group (invasive OC incidence, %)	CA125 threshold (U/ml)	Sensitivity, % (95% CI)	Specificity, % (95% CI)	PPV, % (95% CI)	NPV, % (95% CI)
18- 29 years (0.13%)	≥35	56.5 (34.5; 76.8)	94.8 (94.4; 95.1)	1.3 (0.7; 2.3)	99.9 (99.9; 100)
	≥34	56.5 (34.5; 76.8)	94.4 (94.1; 94.8)	1.3 (0.7; 2.1)	99.9 (99.9; 100)
	≥91	30.4 (13.2; 52.9)	98.8 (98.6; 98.9)	3.0 (1.2; 6.1)	99.9 (99.9; 99.9)
30-39 years (0.14%)	≥35	69.1 (55.2; 80.9)	92.4 (92.1; 92.6)	1.2 (0.9; 1.7)	100 (99.9; 100)
	≥59	50.9 (37.1; 64.6)	97.3 (97.1; 97.5)	2.6 (1.7; 3.7)	99.9 (99.9; 100)
	≥160	29.1 (17.6; 42.9)	99.4 (99.3; 99.5)	6.2 (3.6; 9.9)	99.9 (99.9; 99.9)
40-49 years (0.26%)	≥35	78.7 (72.7; 83.9)	92.0 (91.9; 92.2)	2.5 (2.1; 2.9)	99.9 (99.9; 100)
	≥58	67.4 (60.8; 73.6)	97.2 (97.1; 97.3)	5.8 (4.9; 6.8)	99.9 (99.9; 99.9)
	≥157	48.4 (41.7; 55.2)	99.4 (99.4; 99.5)	17.4 (14.5; 20.7)	99.9 (99.9; 99.9)
Age 50-59 years (0.52%)	≥35	80.5 (76.3; 84.3)	95.7 (76.3; 84.3)	8.8 (7.9; 9.7)	99.9 (99.9; 99.9)
	≥26	84.8 (80.9; 88.2)	91.6 (91.4; 91.8)	5.0 (4.5; 5.5)	99.9 (99.9; 99.9)
	≥57	72.3 (67.7; 76.6)	98.1 (98.0; 98.2)	16.7 (14.9; 18.5)	99.9 (99.8; 99.9)
Age 60-69 years (1.05%)	≥35	86.9 (83.9; 89.5)	95.9 (95.8; 96.1)	18.5 (17.1; 19.9)	99.9 (99.8; 99.9)
	≥22	92.4 (90.0; 94.4)	89.3 (89.0; 89.5)	8.4 (7.7; 9.1)	99.9 (99.8; 99.9)
	≥37	86.6 (83.6; 89.2)	96.2 (96.1; 96.4)	19.7 (18.2; 21.3)	99.9 (99.8; 99.9)
Age 70-79 years (1.32%)	≥35	87.7 (84.6; 90.3)	93.6 (93.4; 93.8)	15.5 (14.2; 16.8)	99.8 (99.8; 99.9)
	≥22	93.5 (91.0; 95.4)	84.7 (84.4; 85.1)	7.6 (6.9; 8.2)	99.9 (99.9; 99.9)
	≥41	86.4 (83.2; 89.2)	94.9 (94.6; 95.1)	18.3 (16.8; 19.9)	99.8 (99.8; 99.8)
Age 80-89 years (1.26%)	≥35	91.4 (87.7; 94.3)	87.3 (86.9; 87.8)	8.7 (7.7; 9.7)	99.9 (99.8; 99.9)
	≥26	92.2 (88.1; 95.1)	81.8 (81.2; 82.3)	6.1 (5.3; 6.9)	99.9 (99.8; 99.9)
	≥58	83.1 (78.0; 87.5)	94.0 (93.6; 94.3)	15.0 (13.2; 16.9)	99.8 (99.7; 99.8)

CA125 = cancer antigen 125, NPV = negative predictive value, OC = ovarian cancer, PPV = positive predictive value

4.6. Ovatools performance to detect invasive ovarian cancer by recorded symptoms

Sub-group	Recorded symptoms*	AUC	Slope	Intercept	O:E	CITL
All ages	Symptoms	0.9601 (0.9565-0.9634)	1.027 (1.0267;1.0273)	-0.0006 (-0.0009;-0.0003)	1.049	0.071
	No symptoms	0.9252 (0.9116-0.9387)	0.925 (0.9248;0.9525)	0.0017 (0.0015;0.0020)	0.761	-0.369
<50 years	Symptoms	0.9070 (0.8761-0.9380)	1.096 (1.0957;1.0963)	-0.0005 (-0.0008;-0.0002)	1.127	0.134
	No symptoms	0.8646 (0.8233-0.9060)	1.011 (1.0107;1.0113)	0.0005 (0.0002;0.0008)	0.769	-0.283
>50 years	Symptoms	0.9600 (0.9529-0.9669)	1.023 (1.0226;1.0234)	-0.0007 (-0.0011;-0.0003)	1.039	0.059
	No symptoms	0.9329 (0.9183-0.9475)	0.947 (0.9466;0.9474)	0.0025 (0.0021;0.0029)	0.759	-0.392

*Symptom of ovarian cancer were taken from the NICE guidelines and included lower abdominal or pelvic pain, lower abdominal or pelvic mass, ascites, bloating, distension, weight loss, appetite loss, fatigue, change in bowel habit, inflammatory bowel syndrome, urinary frequency and urinary urgency. Any coded records in the 90 days prior to CA125 testing that indicated either symptoms or prescriptions that could be used for these symptoms were included.
AUC = area under the curve; CITL = calibration in the large; O:E = observed vs expected outcomes.

Supplement 5: Clinical utility

We report below on the estimated number of women under 90 years who would be referred for further investigation following CA125 in England per year and the number of cases of invasive ovarian cancer (OC) that could be identified using:

(1) Using the Ovatools risk prediction model, where 1-2.9% risk and ≥3% risk triggering primary care ultrasound and direct urgent cancer pathway referral, respectively (**Table S5.1.11.**)

(2) Using integer CA125 thresholds that equate to approximately ≥1% and ≥3% risk of invasive OC by age group to trigger ultrasound and direct cancer referral, respectively. We use age categorisations: 18-29, 30-39, 40-49, 50-59, 60-69, 70-79 and 80-89 years (**Table S5.2.12**)

Utility for both groups is compared to that using current NICE guidelines, in which a CA125 test result ≥35U/ml triggers primary care ultrasound (referred to as "current practice"). For both groups above, we estimate the utility overall, by ages above and below 50 years, and by age group.

Table S5.i. The number of women tested using CA125 in primary care in England in 2022

	Number
Women tested with CA125 in 2022	34,075
GP practices with CA125 records in CPRD	1,009
Average women tested using CA125 per GP practice per year	33.77
Total GP practices in England in 2022[25]	6,422
Total tested with CA125 in England in 2022 under 90 years	216,878
Number with invasive ovarian cancer (incidence 0.62%) in England	1,339
Total population in England in 2022[26]	57,112,500
CA125 tests per 100,000 population per year	380
Number with invasive ovarian cancer / 100,000 population	2.39

The mean number of women who received one or more CA125 tests per GP practice per year in CPRD between 28 February 2021 and 1 March 2022 was calculated (**Table S5.i**). Women with a prior diagnosis of any invasive ovarian cancer were excluded. Based on 34,075 women under 90 years tested with CA125 in one year across 1,009 GP practices in CPRD, this equated to approximately 33.77 women per GP practice per year. With 6,422 GP practices in 2022[25], a total of 216,878 women under 90 years were estimated to have been tested with CA125 in England in 2022. The population in England in 2022 was approximately 57,112,500[26], thus, an estimated 380 women had one or more CA125 tests per 100,000 population in 2022, of which 2.39 had invasive ovarian cancer.

Table S5.ii. The number of women under 90 years tested using CA125 in primary care in England in 2022 by age group

Age group	Population distribution, %	Number tested using CA125 per year	Invasive OC incidence, n (%)
18-29 years	5.40%	11,711	23 (0.13)
30-39 years	11.69%	25,353	55 (0.14)
40-49 years	25.17%	54,588	221 (0.26)
50-59 years	22.91%	49,687	401 (0.52)
60-69 years	16.88%	36,609	603 (1.05)
70-79 years	11.98%	25,982	536 (1.32)
80-89 years	5.97%	12,948	255 (1.26)

Section 5.1. Clinical utility of using Ovatools risk models at 1-2.9% and ≥3% to trigger ultrasound and urgent referral, respectively, compared to current practice.

Table S5.1.1. Distribution of CA125 ≥35U/ml and Ovatools risk among CA125-tested women per year, all ages

CA125 and Ovatools risk distribution for all participants and by invasive ovarian cancer diagnosis		Proportion, %	Number in cohort, total=339,124	Number in England per year, total=216,878
All participants	CA125 <35U/mL	93.20	316,068	202,133
	CA125 ≥35U/mL	6.80	23,056	14,745
	<1% risk	92.05	312,152	199,629
	≥1% risk	7.95	26,972	17,249
	1-3%	5.25	17,806	11,387
	≥3% risk	2.70	9,166	5,862
Participants with invasive ovarian cancer	CA125 ≥35U/mL	84.67	1,773	1,134
	≥1% risk	86.82	1,818	1,163
	≥3% risk	77.55	1,624	1,039
Participants without invasive ovarian cancer	CA125 ≥35U/mL	6.31	21,283	13,611
	≥1% risk	7.46	25,154	16,087
	≥3% risk	2.24	7,542	4,238

Table S5.1.2. Distribution of CA125 ≥35U/ml and Ovatools risk among CA125-tested women per year, 18-29 years

CA125 and Ovatools risk distribution for all participants and by invasive ovarian cancer diagnosis		Proportion, %	Number in cohort, total=18,319	Number in England per year, total=11,711
All participants	CA125 <35U/mL	94.69	17,347	11,090
	CA125 ≥35U/mL	5.31	972	621
	<1% risk	95.39	17,474	11,171
	≥1% risk	4.61	845	540
	1-3%	3.53	647	414
	≥3% risk	1.08	198	127
Participants with invasive ovarian cancer	CA125 ≥35U/mL	56.52	13	8
	≥1% risk	47.83	11	7
	≥3% risk	39.13	9	6
Participants without invasive ovarian cancer	CA125 ≥35U/mL	5.24	959	613
	≥1% risk	4.56	834	533
	≥3% risk	1.03	189	118

Table S5.1.3. Distribution of CA125 ≥35U/ml and Ovatools risk among CA125-tested women per year, 30-39 years

CA125 and Ovatools risk distribution for all participants and by invasive ovarian cancer diagnosis		Proportion, %	Number in cohort, total=39,635	Number in England per year, total=25,353
All participants	CA125 <35U/mL	0.14	55	35
	CA125 ≥35U/mL	99.86	39,580	25,318
	<1% risk	92.27	36,570	23,392
	≥1% risk	7.73	3,065	1,961
	1-3%	97.43	38,617	24,702
	≥3% risk	2.57	1,018	651
Participants with invasive ovarian cancer	CA125 ≥35U/mL	69.09	38	24
	≥1% risk	49.09	27	17
	≥3% risk	23.64	13	8
Participants without invasive ovarian cancer	CA125 ≥35U/mL	7.65	3,027	1,936
	≥1% risk	2.50	991	634
	≥3% risk	0.55	219	135

Table S5.1.4. Distribution of CA125 ≥35U/ml and Ovatools risk among CA125-tested women per year, 40-49 years

CA125 and Ovatools risk distribution for all participants and by invasive ovarian cancer diagnosis		Proportion, %	Number in cohort, total=39,635	Number in England per year, total=25,353
All participants	CA125 <35U/mL	91.86	78,396	50,144
	CA125 ≥35U/mL	8.14	6,948	4,444
	<1% risk	96.82	82,628	52,851
	≥1% risk	3.18	2,716	1,737
	1-3%	2.44	2,084	1,333
	≥3% risk	0.74	632	404
Participants with invasive ovarian cancer	CA125 ≥35U/mL	78.73	174	111
	≥1% risk	66.52	147	94
	≥3% risk	51.13	113	72
Participants without invasive ovarian cancer	CA125 ≥35U/mL	7.96	6,774	4,333
	≥1% risk	3.02	2,569	1,643
	≥3% risk	0.61	519	291

Table S5.1.5. Distribution of CA125 ≥35U/ml and Ovatools risk among CA125-tested women per year, 50-59 years

CA125 and Ovatools risk distribution for all participants and by invasive ovarian cancer diagnosis		Proportion, %	Number in cohort, total= 77,697	Number in England per year, total=49,687
All participants	CA125 <35U/mL	0.52	401	256
	CA125 ≥35U/mL	99.48	77,296	49,430
	<1% risk	95.26	74,012	47,330
	≥1% risk	4.74	3,685	2,357
	1-3%	0.52	401	256
	≥3% risk	99.48	77,296	49,430
Participants with invasive ovarian cancer	CA125 ≥35U/mL	80.55	323	207
	≥1% risk	84.54	339	217
	≥3% risk	72.32	290	185
Participants without invasive ovarian cancer	CA125 ≥35U/mL	4.35	3,362	2,150
	≥1% risk	7.03	5,434	3,475
	≥3% risk	1.58	1,225	679

Table S5.1.6. Distribution of CA125 ≥35U/ml and Ovatools risk among CA125-tested women per year, 60-69 years

CA125 and Ovatools risk distribution for all participants and by invasive ovarian cancer diagnosis		Proportion, %	Number in cohort, total= 57,257	Number in England per year, total=36,609
All participants	CA125 <35U/mL	95.05	54,420	34,795
	CA125 ≥35U/mL	4.95	2,837	1,814
	<1% risk	88.44	50,637	32,376
	≥1% risk	11.56	6,620	4,233
	1-3%	6.93	3,969	2,538
	≥3% risk	4.63	2,651	1,695
Participants with invasive ovarian cancer	CA125 ≥35U/mL	86.90	524	335
	≥1% risk	92.37	557	356
	≥3% risk	86.57	522	334
Participants without invasive ovarian cancer	CA125 ≥35U/mL	4.08	2,313	1,479
	≥1% risk	10.70	6,063	3,877
	≥3% risk	3.76	2,129	1,173

Table S5.1.7. Distribution of CA125 ≥35U/ml and Ovatools risk among CA125-tested women per year, 70-79 years

CA125 and Ovatools risk distribution for all participants and by invasive ovarian cancer diagnosis		Proportion, %	Number in cohort, total= 40,624	Number in England per year, total=25,982
All participants	CA125 <35U/mL	92.53	37,589	24,041
	CA125 ≥35U/mL	7.47	3,035	1,941
	<1% risk	84.86	34,472	22,047
	≥1% risk	15.14	6,152	3,935
	1-3%	9.00	3,656	2,338
	≥3% risk	6.14	2,496	1,596
Participants with invasive ovarian cancer	CA125 ≥35U/mL	87.69	470	301
	≥1% risk	93.66	502	321
	≥3% risk	86.01	461	295
Participants without invasive ovarian cancer	CA125 ≥35U/mL	6.40	2,565	1,641
	≥1% risk	14.09	5,650	3,614
	≥3% risk	5.08	2,035	1,135

Table S5.1.8. Distribution of CA125 ≥35U/ml and Ovatools risk among CA125-tested women per year, 80-89 years

CA125 and Ovatools risk distribution for all participants and by invasive ovarian cancer diagnosis		Proportion, %	Number in cohort, total= 20,248	Number in England per year, total=12,948
All participants	CA125 <35U/mL	87.58	17,734	11,340
	CA125 ≥35U/mL	12.42	2,514	1,608
	<1% risk	81.00	16,400	10,487
	≥1% risk	19.00	3,848	2,461
	1-3%	11.88	2,406	1,539
	≥3% risk	7.12	1,442	922
Participants with invasive ovarian cancer	CA125 ≥35U/mL	90.59	231	148
	≥1% risk	92.16	235	150
	≥3% risk	84.71	216	138
Participants without invasive ovarian cancer	CA125 ≥35U/mL	11.42	2,283	1,460
	≥1% risk	18.07	3,613	2,310
	≥3% risk	6.13	1,226	706

Table S5.1.9. Distribution of CA125 ≥35U/ml and Ovatools risk among CA125-tested women per year, 18-49 years

CA125 and Ovatools risk distribution for all participants and by invasive ovarian cancer diagnosis		Proportion, %	Number in cohort, total= 143,298	Number in England per year, total=91,653
All participants	CA125 <35U/mL	92.33	132,313	84,627
	CA125 ≥35U/mL	7.67	10,985	7,026
	<1% risk	96.80	138,719	88,724
	≥1% risk	3.20	4,579	2,929
	1-3%	2.45	3,517	2,249
	≥3% risk	0.74	1,062	679
Participants with invasive ovarian cancer	CA125 ≥35U/mL	75.25	225	144
	≥1% risk	61.87	185	118
	≥3% risk	45.15	135	86
Participants without invasive ovarian cancer	CA125 ≥35U/mL	7.52	10,760	6,882
	≥1% risk	3.07	4,394	2,810
	≥3% risk	0.65	927	544

Table S5.1.10. Distribution of CA125 ≥35U/ml and Ovatools risk among CA125-tested women per year, 50-89 years

CA125 and Ovatools risk distribution for all participants and by invasive ovarian cancer diagnosis		Proportion, %	Number in cohort, total= 143,298	Number in England per year, total=91,653
All participants	CA125 <35U/mL	93.84	183,755	117,506
	CA125 ≥35U/mL	6.16	12,071	7,719
	<1% risk	88.87	174,038	111,292
	≥1% risk	11.44	22,393	14,320
	1-3%	7.30	14,289	9,137
	≥3% risk	4.14	8,104	5,182
Participants with invasive ovarian cancer	CA125 ≥35U/mL	86.24	1,548	990
	≥1% risk	90.97	1,633	1,044
	≥3% risk	82.95	1,489	952
Participants without invasive ovarian cancer	CA125 ≥35U/mL	5.42	10,523	6,729
	≥1% risk	10.70	20,760	13,275
	≥3% risk	3.41	6,615	3,693

Table S5.1.11. Women investigated further and identified with invasive OC using current practice compared to Ovatools risks 1-2.9% and ≥3% to trigger ultrasound or cancer referral, respectively.

Age group	Age distribution, %	Threshold for primary care ultrasound, U/ml	Ultrasound following CA125, n	Change in USS using Ovatools, n	Urgent referral (risk ≥3%), n	Additional women tested further using Ovatools, n	Invasive OC detected, n	Additional OC detected using Ovatools, n
All ages	NA	CA125 ≥35U/mL	14,745	NA	NA	NA	1,134	29
		Ovatools 1-2.9%	11,387	-3,357	5,862	2,504	1,163	
All ages 18-49 years	42.26	CA125 ≥35U/mL	7,026	NA	NA	NA	144	-26
		Ovatools 1-2.9%	2,249	-4,776	679	-4,097	118	
All ages 50-89 years	57.74	CA125 ≥35U/mL	7,719	NA	NA	NA	990	54
		Ovatools 1-2.9%	9,137	1,418	5,182	6,601	1,044	
18-29 years	5.40	CA125 ≥35U/mL	621	NA	NA	NA	8	-1
		Ovatools 1-2.9%	414	-208	127	-81	7	
30-39 years	11.69	CA125 ≥35U/mL	1,961	NA	NA	NA	24	-7
		Ovatools 1-2.9%	503	-1,458	148	-1,309	17	
40-49 years	25.17	CA125 ≥35U/mL	4,444	NA	NA	NA	111	-17
		Ovatools 1-2.9%	1,333	-3,111	404	-2,707	94	
50-59 years	22.91	CA125 ≥35U/mL	2,357	NA	NA	NA	207	10
		Ovatools 1-2.9%	2,723	366	969	1,335	217	
60-69 years	16.88	CA125 ≥35U/mL	1,814	NA	NA	NA	335	21
		Ovatools 1-2.9%	2,538	724	1,695	2,419	356	
70-79 years	11.98	CA125 ≥35U/mL	1,941	NA	NA	NA	301	20
		Ovatools 1-2.9%	2,338	397	1,596	1,994	321	
80-89 years	5.97	CA125 ≥35U/mL	1,608	NA	NA	NA	148	3
		Ovatools 1-2.9%	1,539	-69	922	853	150	

Section 5.2: Clinical utility using age-group based CA125 levels that approximate 1-2.9% and 3% Ovatools risk to trigger ultrasound and urgent referral, respectively.

Table 5.2.1. The CA125 thresholds used to estimate to trigger ultrasound or cancer referral

Age group	Equivalent CA125 level (U/ml)	
	Ovatools 1-2.9% risk (primary care ultrasound)	Ovatools ≥3% risk (urgent cancer referral)
18-29 years	34-90.9	≥91
30-39 years	59-159.9	≥160
40-49 years	58-156.9	≥157
50-59 years	26-56.9	≥57
60-69 years	22-36.9	≥37
70-79 years	22-40.9	≥41
80-89 years	26-57.9	≥58

Table S5.2.2. Distribution of women with CA125 values above/below the 1% and 3% risk thresholds, all ages

CA125 / risk distribution for all participants and by invasive ovarian cancer diagnosis		Proportion, %	Number in cohort, total=339,124	Number in England per year, total=216,878
All participants	<1% risk*	91.08	308,877	197,534
	≥1% risk*	8.92	30,247	19,344
	1-3%*	6.06	20,550	13,142
	≥3% risk*	2.86	9,697	6,201
Participants with invasive ovarian cancer	≥1% risk*	86.82	1,818	1,163
	≥3% risk*	77.55	1,624	1,039
Participants without invasive ovarian cancer	≥1% risk*	7.46	25,154	16,087
	≥3% risk*	2.24	7,542	4,238
*CA125 thresholds applied by age group as defined in Table S5.2.1				

Table S5.2.3. Distribution of women with CA125 values above/below the 1% and 3% risk thresholds, 18-29 years

CA125 /risk distribution for all participants and by invasive ovarian cancer diagnosis, U/ml (% risk)		Proportion, %	Number in cohort, total=18,319	Number in England per year, total=11,711
All participants	CA125 <34 (<1%)	94.37	17,287	11,052
	CA125 = 34-90.9 (1-3% risk)	4.36	799	511
	CA125 ≥91 (≥3% risk)	1.27	233	149
Participants with invasive ovarian cancer	CA125 ≥34 (≥1% risk)	56.52	13	8
	CA125 ≥91 (≥3% risk)	30.43	7	4
Participants without invasive ovarian cancer	CA125 ≥34 (≥1% risk)	7.46	5.57	1,019
	CA125 ≥91 (≥3% risk)	2.24	1.24	226

Table S5.2.4. Distribution of women with CA125 values above/below the 1% and 3% risk thresholds, 30-39 years

CA125 /risk distribution for all participants and by invasive ovarian cancer diagnosis, U/ml (% risk)		Proportion, %	Number in cohort, total=39,635	Number in England per year, total=25,353
All participants	CA125 <59 (<1%)	97.24	38,541	24,653
	CA125 = 59-159.9 (1-3% risk)	2.11	837	535
	CA125 ≥160 (≥3% risk)	0.65	257	164
Participants with invasive ovarian cancer	CA125 ≥59 (≥1% risk)	50.91	28	18
	CA125 ≥160 (≥3% risk)	29.09	16	10
Participants without invasive ovarian cancer	CA125 ≥59 (≥1% risk)	2.69	1,066	682
	CA125 ≥160 (≥3% risk)	0.61	241	148

Table S5.2.5. Distribution of women with CA125 values above/below the 1% and 3% risk thresholds, 40-49 years

CA125 /risk distribution for all participants and by invasive ovarian cancer diagnosis, U/ml (% risk)		Proportion, %	Number in cohort, total=85,344	Number in England per year, total=54,588
All participants	CA125 <58 (<1%)	97.00	82,784	52,951
	CA125 = 58-156.9 (1-3% risk)	2.28	1,946	1,245
	CA125 ≥157 (≥3% risk)	0.72	614	393
Participants with invasive ovarian cancer	CA125 ≥58 (≥1% risk)	67.42	149	95
	CA125 ≥157 (≥3% risk)	48.42	107	68
Participants without invasive ovarian cancer	CA125 ≥58 (≥1% risk)	2.83	2,411	1,542
	CA125 ≥157 (≥3% risk)	0.60	507	286

Table S5.2.6. Distribution of women with CA125 values above/below the 1% and 3% risk thresholds, 50-59 years

CA125 /risk distribution for all participants and by invasive ovarian cancer diagnosis, U/ml (% risk)		Proportion, %	Number in cohort, total=77,697	Number in England per year, total=49,687
All participants	CA125 <26 (<1%)	91.19	70,849	45,307
	CA125 = 26-56.9 (1-3% risk)	6.57	75,956	3,266
	CA125 ≥57 (≥3% risk)	2.24	1,741	1,113
Participants with invasive ovarian cancer	CA125 ≥26 (≥1% risk)	84.79	340	217
	CA125 ≥57 (≥3% risk)	72.32	290	185
Participants without invasive ovarian cancer	CA125 ≥26 (≥1% risk)	8.42	6,508	4,162
	CA125 ≥57 (≥3% risk)	1.88	1,451	823

Table S5.2.7. Distribution of women with CA125 values above/below the 1% and 3% risk thresholds, 60-69 years

CA125 /risk distribution for all participants and by invasive ovarian cancer diagnosis, U/ml (% risk)		Proportion, %	Number in cohort, total=57,257	Number in England per year, total=36,609
All participants	CA125 <22 (<1%)	88.42	50,625	32,369
	CA125 = 22-36.9 (1-3% risk)	6.95	54,607	2,546
	CA125 ≥37 (≥3% risk)	4.63	2,650	1,694
Participants with invasive ovarian cancer	CA125 ≥22 (≥1% risk)	92.37	557	356
	CA125 ≥37 (≥3% risk)	86.57	522	334
Participants without invasive ovarian cancer	CA125 ≥22 (≥1% risk)	10.72	6,075	3,884
	CA125 ≥37 (≥3% risk)	3.76	2,128	1,172

Table S5.2.8. Distribution of women with CA125 values above/below the 1% and 3% risk thresholds, 70-79 years

CA125 /risk distribution for all participants and by invasive ovarian cancer diagnosis, U/ml (% risk)		Proportion, %	Number in cohort, total=40,624	Number in England per year, total=25,982
All participants	CA125 <22 (<1%)	83.70	34,004	21,748
	CA125 = 22-40.9 (1-3% risk)	10.08	38,097	2,618
	CA125 ≥41 (≥3% risk)	6.22	2,527	1,616
Participants with invasive ovarian cancer	CA125 ≥22 (≥1% risk)	93.47	501	320
	CA125 ≥41 (≥3% risk)	86.38	463	296
Participants without invasive ovarian cancer	CA125 ≥22 (≥1% risk)	15.26	6,119	3,914
	CA125 ≥41 (≥3% risk)	5.15	2,064	1,153

Table S5.2.9. Distribution of women with CA125 values above/below the 1% and 3% risk thresholds, 80-89 years

CA125 /risk distribution for all participants and by invasive ovarian cancer diagnosis, U/ml (% risk)		Proportion, %	Number in cohort, total=20,248	Number in England per year, total=12,948
All participants	CA125 <26 (<1%)	80.84	16,369	10,467
	CA125 = 26-57.9 (1-3% risk)	12.17	18,833	1,576
	CA125 ≥58 (≥3% risk)	6.99	1,415	905
Participants with invasive ovarian cancer	CA125 ≥26 (≥1% risk)	92.16	235	150
	CA125 ≥58 (≥3% risk)	83.14	212	136
Participants without invasive ovarian cancer	CA125 ≥26 (≥1% risk)	18.23	3,644	2,330
	CA125 ≥58 (≥3% risk)	6.02	1,203	693

Table S5.2.10. Distribution of women with CA125 values above/below the 1% and 3% risk thresholds, 18-49 years

CA125 and Ovatools risk distribution for all participants and by invasive ovarian cancer diagnosis		Proportion, %	Number in cohort, total=143,298	Number in England per year, total=91,653
All participants	<1% risk*	96.73	138,612	88,655
	1-3%*	2.50	3,582	2,291
	≥3% risk*	0.77	1,104	706
Participants with invasive ovarian cancer	≥1% risk*	63.55	190	122
	≥3% risk*	43.48	130	83
Participants without invasive ovarian cancer	≥1% risk*	3.14	4,496	2,876
	≥3% risk*	0.68	974	576
*CA125 thresholds applied by age group as defined in Table S5.2.1				

Table S5.2.11. Distribution of women with CA125 values above/below the 1% and 3% risk thresholds, 50-89 years

CA125 and Ovatools risk distribution for all participants and by invasive ovarian cancer diagnosis		Proportion, %	Number in cohort, total=195,826	Number in England per year, total=125,225
All participants	<1% risk*	87.75	171,847	109,891
	≥1% risk*	7.99	15,646	10,005
	1-3%*	4.26	8,333	5,329
	≥3% risk*	2.86	9,697	6,201
Participants with invasive ovarian cancer	≥1% risk*	90.97	1,633	1,044
	≥3% risk*	82.84	1,487	951
Participants without invasive ovarian cancer	≥1% risk*	11.52	22,346	14,290
	≥3% risk*	3.53	6,846	3,842
*CA125 thresholds applied by age group as defined in Table S5.2.1				

Table S5.2.12. The clinical utility of using CA125 thresholds that approximate 1 and 3% risk by age group to trigger ultrasound and cancer referral, respectively, compared to current practice.

Age group	Age distribution, %	Threshold for primary care ultrasound, U/ml (% risk)	Ultrasound following CA125, n	Change in ultrasound using Ovatools, n	Urgent referral (risk ≥3%*), n	Additional tested further using Ovatools, n	Invasive OC detected, n	Additional OC detected using Ovatools, n
All ages	NA	CA125 ≥35	14,745	NA	NA	NA	1,134	32
		1-2.9% risk*	12,296	-2449	6,035	3,586	1,166	
All 18-49 years	42.26	CA125 ≥35	7,026	NA	NA	NA	144	-22
		1-2.9% risk*	2,291	-4735	706	-4029	122	
All 50-89 years	57.74	CA125 ≥35	7,719	NA	NA	NA	990	54
		1-2.9% risk*	10,005	2286	5,329	7,615	1,044	
18-29 years	5.40	CA125 ≥35	621	NA	NA	NA	8	0
		CA125 ≥34 (≥1% risk)	511	-111	149	38	8	
30-39 years	11.69	CA125 ≥35	1,961	NA	NA	NA	24	-6
		CA125 ≥59 (≥1% risk)	535	-1425	164	-1261	18	
40-49 years	25.17	CA125 ≥35	4,444	NA	NA	NA	111	-16
		CA125 ≥58 (≥1% risk)	1,245	-3199	393	-2807	95	
50-59 years	22.91	CA125 ≥35	2,357	NA	NA	NA	207	11
		CA125 ≥26 (≥1% risk)	3,266	909	1113	2,023	217	
60-69 years	16.88	CA125 ≥35	1,814	NA	NA	NA	335	21
		CA125 ≥22 (≥1% risk)	2,546	732	1,694	2,426	356	
70-79 years	11.98	CA125 ≥35	1,941	NA	NA	NA	301	20
		CA125 ≥22 (≥1% risk)	2,618	677	1,616	2,293	320	
80-89 years	5.97	CA125 ≥35	1,608	NA	NA	NA	148	3
		CA125 ≥26 (≥1% risk)	1,576	-32	905	873	150	

*CA125 thresholds equating approximate risk applied by age group as defined in Table S5.2.1

Supplement 6: Sample size calculation

Taking the approach of Riley *et al*. (1), we calculated the required sample size for precise estimation of observed divided by expected cases, the calibration slope, the C-statistic and the net benefit (clinical utility) at a referral threshold of $\geq$3%. Riley *et al.* (1) recommend that the sample size is at least as large as the maximum of the four required sample size figures. The inputs to the calculations were derived from the estimation set in the model development study (2) as follows: (i) Proportion with disease = 0.009, (ii) Mean and variance of the linear predictor in those with disease = -2.09 and 2.52, respectively (iii) Mean and variance of the linear predictor in those without disease = -6.34 and 1.32, respectively, (iv) C-statistic = 0.92, however, we used C = 0.82 to allow for shrinkage, (v) Sensitivity and specificity at a threshold of 3% = 0.721 and 0.967, respectively.

We anticipated the observed versus expected (O/E) statistic would close to 1.0, and to obtain 95% confidence intervals (CIs) on the logarithm which would retransform to approximately +/- 0.05, we stipulate a standard error (SE) of the logarithm of O/E of 0.025. For this, we calculated a sample size of 176,178 subjects for external validation. For the calibration slope, the shape of the linear predictor distribution in those with ovarian cancer (OC) appeared approximately normal in the estimation dataset. We anticipated the slope, and intercept would be approximately 1 and zero, respectively. We required a SE on the slope of 0.025, giving a 95% CI of approximately +/- 0.05. The distribution in those without OC appeared approximately lognormal. Applying these to a simulated population of 1,000,000 subjects with a mixing fraction of 0.009 (see above), we required a sample of 140,800 subjects. For the C-statistic of 0.92, disease prevalence of 0.009 and a required SE of 0.025, we needed 13,648 subjects. For the more conservative C-statistic of 0.82, we required 32,012 subjects. From the sensitivities and specificities above, the standardised net benefit in the estimation set was 0.61 to have a 95% CI extending no further than =/- 0.1, we specify a SE of 0.05. To achieve a SE of at most 0.05, we needed 38,228 subjects. Using the maximum of the four figures, 176,178 subjects was the minimum sample size of the validation set. We repeated calculations for the subgroup of invasive OC with prevalence 0.007 and C-statistic in the estimation set of 0.946. We specified the same required SE. The minimum study sizes arrived at were: O/E: 226,968 subjects; Calibration slope: 39,876 subjects; C-statistic: 14,056 for C=0.94, 36,788 for C=0.84; Net benefit: 43,372. Using the maximum of these, we aimed for a sample size of at least 226,968 subjects. In our initial sample size calculations, we used a standard error of 0.05 instead of 0.025 as stipulated above. Thus, we initially calculated that we required 56,742 subjects as the maximum of the four calculations above. However, this mistake in our calculation was rectified, and the final minimum sample size was calculated as 226,968 subjects as demonstrated above.

References

1. Riley RD, Debray TPA, Collins GS, Archer L, Ensor J, Van Smeden M, et al. Minimum sample size for external validation of a clinical prediction model with a binary outcome. Statistics in Medicine [Internet]. 2021 Aug 30 [cited 2023 Nov 16];40(19):4230–51. Available from: https://onlinelibrary.wiley.com/doi/10.1002/sim.9025

2. Funston G, Abel G, Crosbie EJ, Hamilton W, Walter FM. Could Ovarian Cancer Prediction Models Improve the Triage of Symptomatic Women in Primary Care? A Modelling Study Using Routinely Collected Data. Cancers [Internet]. 2021 Jun 9 [cited 2023 Nov 16];13(12):2886. Available from: https://doi.org/10.3390/cancers13122886

TRIPOD Checklist: Prediction Model Development

Section/Topic	Item	Checklist Item	Page
**Title and abstract**			
Title	1	Identify the study as developing and/or validating a multivariable prediction model, the target population, and the outcome to be predicted.	1
Abstract	2	Provide a summary of objectives, study design, setting, participants, sample size, predictors, outcome, statistical analysis, results, and conclusions.	2
**Introduction**			
Background and objectives	3a	Explain the medical context (including whether diagnostic or prognostic) and rationale for developing or validating the multivariable prediction model, including references to existing models.	2
	3b	Specify the objectives, including whether the study describes the development or validation of the model or both.	3
**Methods**			
Source of data	4a	Describe the study design or source of data (e.g., randomized trial, cohort, or registry data), separately for the development and validation data sets, if applicable.	3
	4b	Specify the key study dates, including start of accrual; end of accrual; and, if applicable, end of follow-up.	3
Participants	5a	Specify key elements of the study setting (e.g., primary care, secondary care, general population) including number and location of centres.	3
	5b	Describe eligibility criteria for participants.	3
	5c	Give details of treatments received, if relevant.	NA
Outcome	6a	Clearly define the outcome that is predicted by the prediction model, including how and when assessed.	4
	6b	Report any actions to blind assessment of the outcome to be predicted.	NA
Predictors	7a	Clearly define all predictors used in developing or validating the multivariable prediction model, including how and when they were measured.	4-5
	7b	Report any actions to blind assessment of predictors for the outcome and other predictors.	NA
Sample size	8	Explain how the study size was arrived at.	6
Missing data	9	Describe how missing data were handled (e.g., complete-case analysis, single imputation, multiple imputation) with details of any imputation method.	NA
Statistical analysis methods	10a	Describe how predictors were handled in the analyses.	NA
	10b	Specify type of model, all model-building procedures (including any predictor selection), and method for internal validation.	4-5
	10d	Specify all measures used to assess model performance and, if relevant, to compare multiple models.	5
Risk groups	11	Provide details on how risk groups were created, if done.	NA
**Results**			
Participants	13a	Describe the flow of participants through the study, including the number of participants with and without the outcome and, if applicable, a summary of the follow-up time. A diagram may be helpful.	NA
	13b	Describe the characteristics of the participants (basic demographics, clinical features, available predictors), including the number of participants with missing data for predictors and outcome.	6-7
Model development	14a	Specify the number of participants and outcome events in each analysis.	6-7
	14b	If done, report the unadjusted association between each candidate predictor and outcome.	NA
Model specification	15a	Present the full prediction model to allow predictions for individuals (i.e., all regression coefficients, and model intercept or baseline survival at a given time point).	7-8<br>Supplement 2
	15b	Explain how to the use the prediction model.	4-5
Model performance	16	Report performance measures (with CIs) for the prediction model.	6-7
**Discussion**			
Limitations	18	Discuss any limitations of the study (such as nonrepresentative sample, few events per predictor, missing data).	13
Interpretation	19b	Give an overall interpretation of the results, considering objectives, limitations, and results from similar studies, and other relevant evidence.	11-12
Implications	20	Discuss the potential clinical use of the model and implications for future research.	12
**Other information**			
Supplementary information	21	Provide information about the availability of supplementary resources, such as study protocol, Web calculator, and data sets.	13
Funding	22	Give the source of funding and the role of the funders for the present study.	13

We recommend using the TRIPOD Checklist in conjunction with the TRIPOD Explanation and Elaboration document.

Supplement 8: Applying the inclusion and exclusion criteria

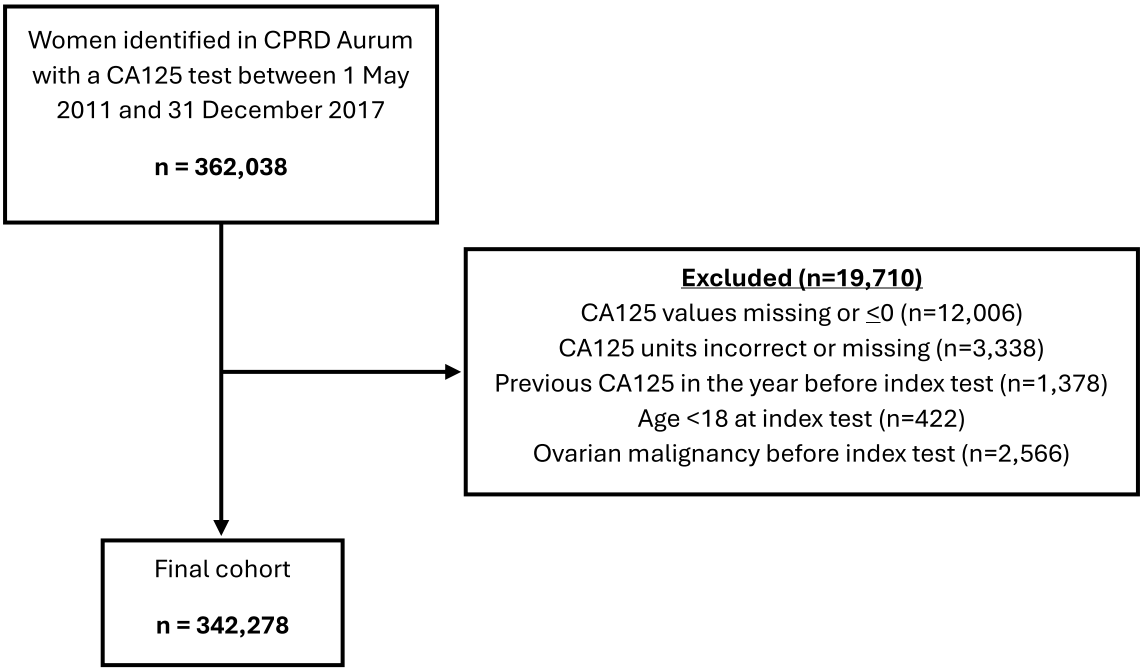

Flow diagram illustrating the selection of the study cohort and application of exclusion criteria.

Supplement 9: Tumour behaviour and morphology

Histological types		Participants with any ovarian cancer, n (%)
Borderline ovarian tumours		512 (19.3)
Invasive ovarian tumours		2143 (80.7)
Epithelial	All epithelial	2,447 (92.2)
	Clear cell	126 (4.8)
	Endometrioid	164 (6.2)
	Mucinous	384 (14.5)
	Serous	1,313 (49.5)
	Other	122 (4.6)
	Unknown	338 (12.7)
Non-epithelial		99 (3.7)
Unknown or other		109 (4.1)

Supplement 10: Additional diagnostic accuracy metrics

Table S10.1. Diagnostic of accuracy of using CA125 thresholds equating to ~1% and ~3% risk of invasive ovarian cancer, compared to standard practice (CA125 ≥35U/ml)

Sub-group (invasive ovarian cancer incidence)	CA125 threshold	Sensitivity, % (95% CI)	Specificity, % (95% CI)	PPV, % (95% CI)	NPV, % (95% CI)
All ages 18-89 years	≥35	84.9 (83.8; 86.4)	93.6 (93.5; 93.6)	7.7 (7.3; 8.0)	99.9 (99.9; 99.9)
	Applied by age category	87.7 (86.2; 89.1)	91.6 (91.5; 91.7)	6.1 (5.8; 6.3)	99.9 (99.9; 99.9)
		78.0 (76.1; 79.7)	97.6 (97.6; 97.6)	16.8 (16.1; 17.6)	99.9 (99.8; 99.9)
Age 18-49 years (0.21%)	≥35	75.3 (70.0; 80.0)	92.5 (92.3; 92.6)	2.0 (1.8; 2.3)	99.9 (99.9; 100)
	≥46	67.9 (62.3; 73.2)	95.8 (95.7; 95.9)	3.2 (2.8; 3.7)	99.9 (99.9; 99.9)
	≥123	48.8 (43.0; 54.6)	99.1 (99.1; 99.2)	10.7 (9.1; 12.5)	99.9 (99.9; 99.9)
All ages 50-89 years (0.92%)	≥35	86.2 (84.6; 87.8)	94.6 (94.5; 94.7)	12.8 (12.2; 13.4)	99.9 (99.8; 99.9)
	Applied by age category	91.0 (89.6; 92.3)	88.5 (88.3; 88.6)	6.8 (6.5; 7.1)	99.9 (99.9; 99.9)
		82.8 (81.0; 84.6)	96.5 (96.4; 96.6)	17.8 (17.0; 18.7)	99.8 (99.8; 99.9)
Age 50-59 years (0.52%)	≥35	80.5 (76.3; 84.3)	95.7 (76.3; 84.3)	8.8 (7.9; 9.7)	99.9 (99.9; 99.9)
	≥26	84.8 (80.9; 88.2)	91.6 (91.4; 91.8)	5.0 (4.5; 5.5)	99.9 (99.9; 99.9)
	≥57	72.3 (67.7; 76.6)	98.1 (98.0; 98.2)	16.7 (14.9; 18.5)	99.9 (99.8; 99.9)
Age 60-69 years (1.05%)	≥35	86.9 (83.9; 89.5)	95.9 (95.8; 96.1)	18.5 (17.1; 19.9)	99.9 (99.8; 99.9)
	≥22	92.4 (90.0; 94.4)	89.3 (89.0; 89.5)	8.4 (7.7; 9.1)	99.9 (99.8; 99.9)
	≥37	86.6 (83.6; 89.2)	96.2 (96.1; 96.4)	19.7 (18.2; 21.3)	99.9 (99.8; 99.9)
Age 70-79 years (1.32%)	≥35	87.7 (84.6; 90.3)	93.6 (93.4; 93.8)	15.5 (14.2; 16.8)	99.8 (99.8; 99.9)
	≥22	93.5 (91.0; 95.4)	84.7 (84.4; 85.1)	7.6 (6.9; 8.2)	99.9 (99.9; 99.9)
	≥41	86.4 (83.2; 89.2)	94.9 (94.6; 95.1)	18.3 (16.8; 19.9)	99.8 (99.8; 99.8)
Age 80-89 years (1.26%)	≥35	90.6 (88.6; 93.9)	88.6 (88.1; 89.0)	9.2 (8.1; 10.4)	99.9 (99.8; 99.9)
	≥26	92.2 (88.1; 95.1)	81.8 (81.2; 82.3)	6.1 (5.3; 6.9)	99.9 (99.8; 99.9)
	≥58	83.1 (78.0; 87.5)	94.0 (93.6; 94.3)	15.0 (13.2; 16.9)	99.8 (99.7; 99.8)
CA125 = cancer antigen 125, CI = confidence interval; NPV = negative predictive value; PPV = positive predictive value					

Table S10.2: The diagnostic accuracy of Ovatools at >1% and >3% risk by early and late-stage cancer, compared to using CA125 >35U/ml

Outcome (incidence)	CA125/Ovatools threshold	Sensitivity, % (95% CI)	Specificity, % (95% CI)	PPV, % (95% CI)	NPV, % (95% CI)
Early-stage invasive OC (n=580, 0.17%)	CA125 ≥35U/mL	66.7 (62.7; 70.6)	93.6 (93.5; 93.7)	1.8 (1.6; 1.9)	99.9 (99.9; 99.9)
	≥1%	70.7 (66.8; 74.4)	92.5 (92.4; 92.5)	1.6 (1.4; 1.7)	99.9 (99.9; 100)
	≥3%	51.7 (47.6; 55.9)	97.8 (97.7; 97.8)	3.8 (3.4; 4.2)	99.9 (99.9; 99.9)
Late-stage invasive OC (n=1247, 0.37%)	CA125 ≥35U/mL	93.3 (91.8; 94.7)	93.6 (93.5; 93.7)	5.1 (4.8; 5.4)	100 (100; 100)
	≥1%	94.6 (93.2; 95.8)	92.5 (92.4; 92.5)	4.4 (4.2; 4.7)	100 (100; 100)
	≥3%	89.6 (87.7; 91.2)	97.8 (97.7; 97.8)	12.8 (12.1; 13.5)	100 (100; 100)

Table S10.3: The diagnostic accuracy of Ovatools at ≥1% and ≥3% risk to detect any ovarian cancer including borderline tumours, compared to using CA125 ≥35U/ml

Sub-group (ovarian cancer incidence %)	CA125/Ovatools threshold	Sensitivity, % (95% CI)	Specificity, % (95% CI)	PPV, % (95% CI)	NPV, % (95% CI)
All women (0.78)	CA125 ≥35U/mL	78.6 (77.0;80.2)	93.6 (93.5;93.7)	8.8 (8.4;9.2)	99.8 (99.8;99.8)
	≥1%	81.1 (79.5; 82.5)	92.5 (92.4;92.5)	7.7 (7.4; 8.1)	99.9 (99.8;99.9)
	≥3%	69.3 (67.5; 71.1)	97.8 (97.7; 97.8)	19.5 (18.7; 20.3)	99.8 (99.7; 99.8)
	≥1%	88.0 (86.5; 89.3)	89.2 (89.0; 89.3)	8.1 (7.8; 8.5)	99.9 (99.8; 99.9)
	≥3%	77.8 (76.0; 79.5)	96.6 (96.5; 96.7)	19.9 (19.1; 20.8)	99.7 (99.7; 99.8)
